# Obligatory roles of dopamine D1 receptors in the dentate gyrus in antidepressant actions of a selective serotonin reuptake inhibitor, fluoxetine

**DOI:** 10.1038/s41380-018-0316-x

**Published:** 2018-12-10

**Authors:** Takahide Shuto, Mahomi Kuroiwa, Naoki Sotogaku, Yukie Kawahara, Yong-Seok Oh, Jin-Hyeok Jang, Chang-Hoon Shin, Yoshinori N. Ohnishi, Yuuki Hanada, Tsuyoshi Miyakawa, Yong Kim, Paul Greengard, Akinori Nishi

**Affiliations:** 1 Department of Pharmacology, Kurume University School of Medicine, Kurume, Fukuoka 830-0011 Japan; 2 Laboratory of Molecular and Cellular Neuroscience, The Rockefeller University, New York, NY 10065 USA; 3 Department of Brain-Cognitive Sciences, Daegu-Gyeongbuk Institute of Science and Technology (DGIST), Hyeonpung-myeon, Dalseong-gun, Daegu 42988, Republic of Korea; 4 Division of Systems Medical Science, Institute for Comprehensive Medical Science, Fujita Health University, Toyoake, Aichi 470-1192 Japan

**Keywords:** Neuroscience, Cell biology

## Abstract

Depression is a leading cause of disability. Current pharmacological treatment of depression is insufficient, and development of improved treatments especially for treatment-resistant depression is desired. Understanding the neurobiology of antidepressant actions may lead to development of improved therapeutic approaches. Here, we demonstrate that dopamine D1 receptors in the dentate gyrus act as a pivotal mediator of antidepressant actions in mice. Chronic administration of a selective serotonin reuptake inhibitor (SSRI), fluoxetine, increases D1 receptor expression in mature granule cells in the dentate gyrus. The increased D1 receptor signaling, in turn, contributes to the actions of chronic fluoxetine treatment, such as suppression of acute stress-evoked serotonin release, stimulation of adult neurogenesis and behavioral improvement. Importantly, under severely stressed conditions, chronic administration of a D1 receptor agonist in conjunction with fluoxetine restores the efficacy of fluoxetine actions on D1 receptor expression and behavioral responses. Thus, our results suggest that stimulation of D1 receptors in the dentate gyrus is a potential adjunctive approach to improve therapeutic efficacy of SSRI antidepressants.

## Introduction

Depression is one of the most common psychiatric disorders, but the etiology of depression is not fully understood [1–3]. Selective serotonin reuptake inhibitors (SSRIs) are widely used as antidepressants, but SSRIs show limited efficacy and delayed responses [1]. In addition, one-third of depressive patients failed to achieve remission after established antidepressant treatment [4]. Further understanding of the neurobiology of depression and antidepressant actions could lead to the development of novel therapeutic approaches for treatment-resistant depression.

Clinical and animal studies indicate that depression is associated with hippocampal dysfunction [5]. In animal models of depression, chronic stress and antidepressant treatment induce molecular and cellular changes in hippocampal neurons [2, 5]. Altered hippocampal function may affect the activity of hippocampal circuits, which are connected to brain regions (such as prefrontal cortex, amygdala, nucleus accumbens and ventral tegmental area) involved in emotional processes [6–8]. In the hippocampal circuits, the dentate gyrus receives excitatory inputs from the entorhinal cortex and functions as the main gateway to the hippocampus. The dentate gyrus has been considered as one of the therapeutic targets of antidepressants because it is involved in mood control and antidepressant-induced adult neurogenesis occurs in the subgranular zone (SGZ) of the dentate gyrus [9, 10].

Dopamine has been implicated in the pathophysiology of depression, and mediates in part the actions of currently available antidepressants [11]. Previous studies showed that chronic administration of fluoxetine (an SSRI) induces the increased expression of dopamine D1 receptors and the immature properties in dentate granule cells, which are called dematuration [12, 13]. Although blockade of D1 receptors was reported to reverse the effects of antidepressants on depression-like behaviors [14–16], the mechanism underlying involvement of D1 receptors has remained unknown. Here we further investigated roles of D1 receptors selectively induced in the dentate gyrus by antidepressants. Biochemical and behavioral analyses revealed that the increase in D1 receptor signaling in mature granule cells in the dentate gyrus was essential for the antidepressant actions. Our results raise the possibility that the D1 receptor in granule cells may be a therapeutic target to enhance antidepressant actions.

## Materials and methods

### Animals

Male C57BL/6N mice were purchased from Japan SLC (Shizuoka, Japan). Mice were housed 2–4 per cage and were maintained on a 12-h light/dark cycle (lights on from 07:00 to 19:00 hours) with access to food and water ad libitum. All mouse handlings were in accordance with the Guide for the Care and Use of Laboratory Animals as adopted by the U.S. National Institutes of Health, and approved by the Institutional Animal Care and Use Committee of Kurume University School of Medicine and of DGIST. All efforts were made to minimize the number of animals. BAC transgenic [*Drd1*]-EGFP (X60) and [*Drd1*]-Cre (EY262) mice were obtained from GENSAT (The Rockefeller University) [17].

### Drug administration

Male C57BL/6N mice at 10 weeks of age were treated with fluoxetine chronically by subcutaneous implantation of matrix-driven delivery pellets (Innovative Research of America, Sarasota, FL), which consistently release fluoxetine at a rate of 15 mg/kg/day for 14 days. In the placebo group, the mice received subcutaneous implantation of pellets containing only matrix for the same period. For the analysis of mRNA expression along the dorsoventral axis, fluoxetine was dissolved in the drinking water and orally applied at a dose of 22 mg/kg/day for 14 days [13].

### Preparation of dentate gyrus slices and immunoblotting

Coronal slices (350 μm) were prepared from the brain between −1.4 and −3.8 mm anterior to bregma as described [18]. The regions of the dentate gyrus were dissected from the slices. Dentate gyrus slices untreated or treated with (±)-SKF81297 (Sigma-Aldrich, St. Louis, MO) were used to determine the levels of mRNAs, proteins, and protein phosphorylation [18]. Antibodies used for immunoblotting are listed in Supplementary Table 1a. In the analysis of mRNA expression along the dorsoventral axis, mRNA levels were quantified in three parts of the dissected dentate gyrus [19]: dorsal, intermediate, and ventral parts.

### Quantitative real-time PCR

Total RNA was isolated from microdissected brain tissues using Sepasol RNA I Super G (Nacalai Tesque, Kyoto, Japan). After genomic DNA cleaning, cDNA was obtained by total RNA reverse transcription using a QuantiTect Reverse Transcription Kit (Qiagen, Valencia, CA). Real-time PCR was performed using LightCycler 480 System II (Roche, Basel, Switzerland) with QuantiFast SYBR Green PCR Master Mix (Qiagen, Valencia, CA) or LightCycler 480 SYBR Green I Master (Roche, Basel, Switzerland). The primer sequences and the PCR protocols are listed in Supplementary Table 2. The relative mRNA levels of individual samples were calculated with the comparative Ct method (2^−^^∆∆Ct^).

### Immunohistochemistry of [*Drd1*]-EGFP mice and quantitative analysis of EGFP-positive neurons

Immunohistochemical analyses were performed using C57BL/6J and [*Drd1*]-EGFP mice treated with or without fluoxetine for 2 weeks as described [20]. Antibodies used for the immunohistochemistry are listed in Supplementary Table 1b.

Quantification of EGFP-positive cells and relative fluorescence intensity were given after background correction using the signal intensity from wild-type, nontransgenic mice. [*Drd1*]-EGFP-positive cells were counted manually on digitized images in each subregion of the hippocampus. For the analyses of fluorescence intensity, the average fluorescent intensity was measured using ImageJ [21] by outlining the region of interest (ROI) for each subregion of the hippocampus and entorhinal cortex. The mean value of individual subregion was divided by the value of ROI area size.

### Neurogenesis assay

The neurogenic effect of chronic fluoxetine treatment in combination with D1 receptor agonist/antagonist was assessed as described [22]. Briefly, the mice were labeled with BrdU solution (200 mg/kg) for 2 h prior to sacrifice by perfusion. Preparation of brain sections and immunostaining were done as described above, and stereological quantitation of BrdU-positive cells was performed as described [22]. Briefly, serial coronal sections (40 µm) were cut through the entire hippocampus in its rostrocaudal extension on a cryostat. Every sixth section throughout the hippocampus was processed for BrdU immunohistochemistry. An experimenter blinded to experimental conditions counted all BrdU-labeled cells in the granule cell layer and the SGZ of the dentate gyrus in the total 12 sections from the individual mouse. The total number of BrdU-labeled cells per section was determined and multiplied by 6 to obtain the total number of cells per dentate gyrus.

### Surgery and brain dialysis

Microdialysis was performed with an I-shaped cannula. Microdialysis probes were implanted in the unilateral dentate gyrus (exposed length 1.0 mm) under pentobarbital (50 mg/kg i.p.) and xylazine (8 mg/kg i.p.) anesthesia and local application of 10% lidocaine. The coordinates of the implantation were A/P −1.7 mm, L/M 1.88 mm, V/D −2.8 mm from the bregma and dura at an angle of 32° in the coronal plane. After surgery, the mice were housed individually in plastic cages (30 × 30 × 40 cm).

Microdialysis experiments were conducted 24–48 h after implantation of the probe, as previously described [23]. Mice in which dialysis probes were misplaced were not included in the data analysis.

### Virus for D1 receptor overexpression

In order to overexpress D1 receptors, adeno-associated virus (AAV) vectors expressing *Drd1* fused with red fluorescent protein (mCherry) under the control of the *EF1α promoter* (*AAV9-EF1α-DIO-Drd1-P2A-mCherry-WPRE*) were stereotaxically injected into the dentate gyrus of [*Drd1*]-Cre mice with similar coordinates to microdialysis probe implantation (A/P −1.7 mm, L/M 1.88 mm, V/D −2.15 mm and angle 32°). *AAV9-EF1α-DIO-Drd1-P2A-mCherry-WPRE* was custom made by the University of North Carolina Vector Core (Chapel Hill, NC). *Drd1* was cloned from a cDNA library obtained from mRNA of the C57BL/6N mouse brain, and integrated into *AAV9-EF1α-DIO-P2A-mCherry-WPRE-hGH vector*. The control virus, *AAV9-EF1α-DIO-eYFP-WPRE-hGH*, was purchased from the University of Pennsylvania Viral Core (Philadelphia, PA).

### Behavioral experiments

#### Experimental schedule 1: regular stress procedure

Male C57BL/6N mice at exactly 8 weeks old were subjected to restraint stress (2 h/day) for 2 weeks (day 1−day 14), in which mice were individually placed in a 50 ml conical tube with breathing holes [24, 25]. On day 15, the pellet releasing fluoxetine at a rate of 15 mg/kg/day was implanted, and then the mice were treated with fluoxetine for 14 days (day 15−day 28). On day 29, after food removal for 24 h in their home cage, the novelty-suppressed feeding test (NSFT) was performed as described [20, 26], and the latency to the first feeding episode was recorded for 300 s [20]. After behavioral tests, the mice were sacrificed by decapitation and brain regions were dissected from coronal slices and used for mRNA analyses.

#### Experimental schedule 2: severe stress procedure

The mice were singly housed in the home cage without bedding materials, and were subjected to restraint stress under the supine position (4 h/day) for 4 weeks (day 1−day 28) [27]. On day 15, the fluoxetine pellet (15 mg/kg/day) was implanted, and the mice were treated with fluoxetine for 14 days (day 15−day 28). In addition, R(+)-SKF81297 was administered intraperitoneally at a dose of 1.5 mg/kg once a day during the last 5 days (day 24−day 28) of fluoxetine treatment. Since R(+)-SKF81297 at a high dose (3.0 mg/kg i.p.) induced behavioral seizures in fluoxetine-treated mice, R(+)-SKF81297 at a dose of 1.5 mg/kg was used. On day 29, the NSFT was conducted first, and then the tail suspension test (TST) [20] and measurements of locomotor activity [28] were conducted with the same cohort of mice. In the TST, mice climbing their tails up to the horizontal-suspension bar were excluded from data [29, 30]. After behavioral tests, brain regions were collected for mRNA and/or protein analyses.

#### Experimental schedule 3: D1 receptor overexpression in the dentate gyrus

For stereotaxic surgeries, mice were positioned in a small-animal stereotaxic instrument (RWD Life Science, San Diego, CA). 0.5 μl of AAV vectors were infused bilaterally at a rate of 0.1 μl/min into the dentate gyrus of [*Drd1*]-Cre mice (bregma coordinates: anterior/posterior, −1.7 mm; medial/lateral, 1.88 mm; dorsal/ventral, 2.15 mm; 32° angle). Four weeks after the virus injection, locomotor activities of the mice were measured first, and then NSFT and TST were performed.

#### Seizure counts

Mice were treated with placebo or fluoxetine (15 mg/kg/day) pellets for 14 days. On day 14, each mouse was isolated from other mice, placed in a transparent cage, and received a challenge injection of R(+)-SKF81297 (1.5 or 3.0 mg/kg i.p.) or saline. After the challenge injection, the mice were recorded by a digital video camera for 60 min, and seizure intensities were analyzed. Each seizure intensity was characterized as follows [31]; phase 1 = sustained immobility/rigidity; phase 2 = rearing with forepaw myoclonus; phase 3 = generalized clonus; phase 4 = tonic-clonic seizure or rapid jumping and wild running.

### Statistical analysis

The data are displayed as mean ± SEM. The significance of differences within each group was determined with one-way ANOVA followed by Neuman−Keuls post hoc test. When two groups were compared, paired or unpaired Student’s *t* test or two-way ANOVA followed by Bonferroni post hoc test was used. The analyses were performed using Prism 5.0 software (GraphPad, San Diego, CA, USA). For analyses of microdialysis data (Fig. 2c−e, Supplemental Figs. 4, 5b), all values were expressed as a percentage of the basal values (100%), obtained as the average of three stable baseline samples. The values obtained after novelty stress were compared with the basal values using mixed linear models with the measurement time as a covariate, and Bonferroni’s correction was applied for multiple comparisons using the SAS MIMED procedure (Version 9.4, SAS Institute, Cary, NC, USA). Repeated measures two-way ANOVA were used to compare the experimental groups (JMP Pro, SAS Institute). *p* < 0.05 was considered to be significant. Details of the statistical analysis are listed in Supplementary Table 3.

## Results

### Chronic fluoxetine treatment induces the expression of D1 receptors in granule cells of the dentate gyrus

**Fig. 1:**
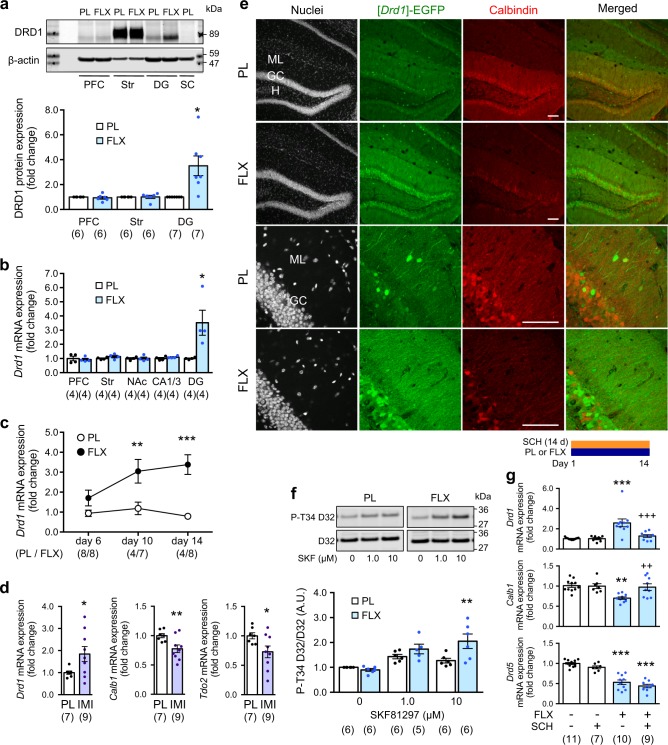
Effect of chronic antidepressant treatment on dopamine D1 receptor expression and signaling in the dentate gyrus. **a** D1 receptor protein (DRD1) expression in the prefrontal cortex (PFC), dorsal striatum (Str), dentate gyrus (DG), and spinal cord (SC) in fluoxetine (FLX; 15 mg/kg/day for 14 days) or placebo (PL) pellet-treated mice. 60 μg (prefrontal cortex, dentate gyrus, and spinal cord) or 6 μg (striatum) of samples were analyzed. Typical immunoblots are shown with quantitation. DRD1 expression is normalized with β-actin. Data represent mean ± SEM. *p *<* 0.05 vs. the placebo group; paired Student’s *t* test. **b** *Drd1* mRNA expression in various brain regions in fluoxetine or placebo pellet-treated mice. NAc nucleus accumbens. Data represent mean ± SEM. *p *<* 0.05 vs. the placebo group; Student’s *t* test. **c** Time course of fluoxetine effects on *Drd1* mRNA expression in the dentate gyrus. Data represent mean ± SEM. **p *<* 0.01, ***p *<* 0.001 vs. the placebo group; two-way ANOVA and Bonferroni post hoc test. **d** mRNA expression of *Drd1*, calbindin-D28K (*Calb1*) and *Tdo2* in the dentate gyrus in imipramine (IMI; 10 mg/kg/day for 14 days) or placebo (PL) pellet-treated mice. Data represent mean ± SEM. *p < 0.05, **p < 0.01 vs. the placebo group; Student’s *t* test. Data in (**a**−**d**) are normalized and represented as fold changes by fluoxetine or imipramine. **e** Immunofluorescence signal of a nuclear dye DraQ5, [*Drd1*]-EGFP and Calbindin-D28K in the dentate gyrus from [*Drd1*]-EGFP mice treated with the fluoxetine or placebo pellet. ML molecular layer, GC granule cell layer, H hilus. Scale bars, 100 µm. **f** Effects of a D1 receptor agonist, (±)-SKF81297 (1 and 10 µM), on PKA-mediated DARPP-32 phosphorylation in slices of the dentate gyrus from mice treated with the fluoxetine or placebo pellet. Typical immunoblots for detection of phospho-Thr34 and total DARPP-32 are shown with quantitation. Data represent mean ± SEM. **p *<* 0.01 vs. the placebo group; two-way ANOVA and Bonferroni post hoc test. **g** Effect of D1 receptor blockade with a specific antagonist, SCH23390 (SCH; 0.1 mg/kg/day i.p. for 14 days), on fluoxetine-induced changes in the gene expression of *Drd1*, *Calb1* and *Drd5* in the dentate gyrus. Data represent mean ± SEM. **p *<* 0.01, ***p *<* 0.001 vs. the fluoxetine (−)/SCH23390 (−) group; ^++^*p* *<* 0.01, ^+++^*p* *<* 0.001 vs. the fluoxetine (+)/SCH23390 (−) group; one-way ANOVA and Newman−Keuls post hoc test. The number of mice is indicated in parentheses under each experimental condition

Chronic fluoxetine treatment (15 mg/kg/day for 14 days) induced increases in Drd1 protein and mRNA only in the dentate gyrus, but not in other brain regions nor in different hippocampal regions (Fig. 1a, b). Chronic fluoxetine treatment also induced a decrease in mRNA expression of markers for mature granule cells in the dentate gyrus including calbindin (*Calb1*), desmoplakin (*Dsp*), tryptophan-2,3-dioxygenase (*Tdo*2), and interleukin-1 receptor (*Il1r1*) (Supplementary Figure 1a-e), as previously reported [13]. In addition, chronic fluoxetine treatment did not affect the mRNA expression of D2-type receptors (*Drd2*, *Drd3* or *Drd4*) (Supplementary Figure 1f), but decreased the mRNA expression of *Drd5* (Supplementary Figure 1f, Fig. 1g). In the analysis of time course, the induction of *Drd1* mRNA expression required at least 10 days of fluoxetine treatment (Fig. 1c), suggesting that the *Drd1* induction in the dentate gyrus is caused by chronic, but not acute, administration of fluoxetine. Analyses along the dorsoventral axis revealed that chronic fluoxetine treatment increased *Drd1* mRNA both in the dorsal and ventral parts of the dentate gyrus, although the expression level of *Drd1* mRNA was lower in the ventral part than in the dorsal part (Supplementary Figure 1g). Notably, chronic treatment with imipramine, a tricyclic antidepressant, also induced an increase in *Drd1* mRNA and a decrease in mRNAs for mature granule cell markers (*Calb1* and *Tdo2*) (Fig. 1d).

Histological analyses using *Drd1 promoter*-driven EGFP reporter mice ([*Drd1*]-EGFP mice) revealed the neuronal cell type where *Drd1* gene is induced in response to chronic fluoxetine treatment. In agreement with a previous report [32, 33], a baseline fluorescence signal of [*Drd1*]-EGFP was detected in a subset of granule cells and presumably GABAergic interneurons in the hilus and molecular layer of the dentate gyrus and the CA1 in placebo-treated mice (Fig. 1e, Supplementary Figure 2). Chronic fluoxetine treatment induced a drastic increase of fluorescence signal mainly in the granule cell layer and the molecular layer, of which the EGFP signal is mainly derived from dendritic arbors of the granule cells (Fig. 1e, Supplementary Figure 3). In addition, we observed a reduced expression of calbindin in mature granule cells, indicating an inverse correlation between D1 receptor and calbindin expression (Fig. 1e). We also examined the possible expression of [*Drd1*]-EGFP at multiple stages of adult neurogenesis in the dentate gyrus. Chronic fluoxetine treatment increased immunofluorescence signal robustly in NeuN-positive mature granule cells and weakly in doublecortin-positive neuroblasts, but neither in Ki-67-positive neural progenitors nor calretinin-positive immature neurons (Supplementary Figure 2).

[*Drd1*]-EGFP expression is also detected in sparse GABAergic interneurons in the molecular layer of the dentate gyrus and the CA subregion (Fig. 1e, Supplementary Figure 3). However, the number of EGFP-positive interneurons and their fluorescence intensity in each subregion were unaffected by chronic fluoxetine treatment (Supplementary Figure 3c, d). Furthermore, contribution of the perforant path to the increased EGFP signal in the molecular layer of the dentate gyrus seems negligible, because [*Drd1*]-EGFP was detected in layer VI neurons of the entorhinal cortex, but rarely in layer II neurons that make direct projection to the dentate gyrus as the perforant path (Supplementary Figure 4). These results altogether indicate that chronic fluoxetine induces D1 receptor expression mainly in mature granule cells.

### Chronic fluoxetine treatment enhances D1 receptor signaling in the dentate gyrus

Dopamine and cyclic AMP-regulated phosphoprotein of M_*r*_ 32,000 (DARPP-32) is a key regulator of PKA signaling, and its role in dopaminergic neurotransmission is extensively characterized in the striatum [34]. DARPP-32 is expressed in granule cells of the dentate gyrus, although its expression level in the dentate gyrus is much lower than that in the striatum (12.8 ± 2.8% of striatal expression) (Supplementary Figure 5a). Treatment of dentate gyrus slices with a D1 receptor agonist, (±)-SKF81297 (1 and 10 µM), increased the level of phospho-Thr34 DARPP-32 by ~1.5-fold. The effect of (±)-SKF81297 on DARPP-32 Thr34 phosphorylation was enhanced by chronic fluoxetine treatment (Fig. 1f). Although chronic fluoxetine treatment did not affect the basal level of phospho-Thr34 DARPP-32, it significantly reduced the basal level of phospho-Ser97 DARPP-32 (the CK2-site) (Supplementary Figure 5b). Because dephosphorylation at Ser97 is associated with nuclear translocation of DARPP-32 and its involvement in epigenetic regulation and gene transcription [35], chronic fluoxetine-induced modulation of genes including *Drd1* is possibly mediated by nuclear DARPP-32. The effects of SKF81297 on the phosphorylation of AMPA receptor GluA1 subunit at Ser845 and ERK2 [36], downstream targets of PKA signaling, were also enhanced by chronic fluoxetine treatment (Supplementary Figure 5c, d). The results suggest that chronic fluoxetine-induced D1 receptors potentiate cAMP/PKA signaling via its intrinsic coupling to Gα_s_ protein and adenylyl cyclase, and modulate both cytoplasmic and nuclear events in granule cells of the dentate gyrus.

### The increase of D1 receptors by chronic fluoxetine treatment requires activation of D1 receptor signaling

We next evaluated the contribution of D1 receptor signaling to the fluoxetine-induced changes in gene expression. The mRNA expression of *Drd1*, *Calb1*, and *Drd5* was examined in the presence or absence of D1 receptor blockade (Fig. 1g). Chronic administration of a D1 receptor antagonist, SCH23390 (0.1 mg/kg/day i.p. for 14 days), abolished the increase of *Drd1* mRNA and the decrease of *Calb1* mRNA, but not the decrease of *Drd5* mRNA, in response to chronic fluoxetine treatment. These results suggest that D1 receptor signaling is required for fluoxetine actions such as modulation of *Drd1* and *Calb1* gene expressions in the dentate gyrus.

### Role of D1 receptors in regulating basal level and novelty stress-induced release, of serotonin (5-HT) in the dentate gyrus

**Fig. 2:**
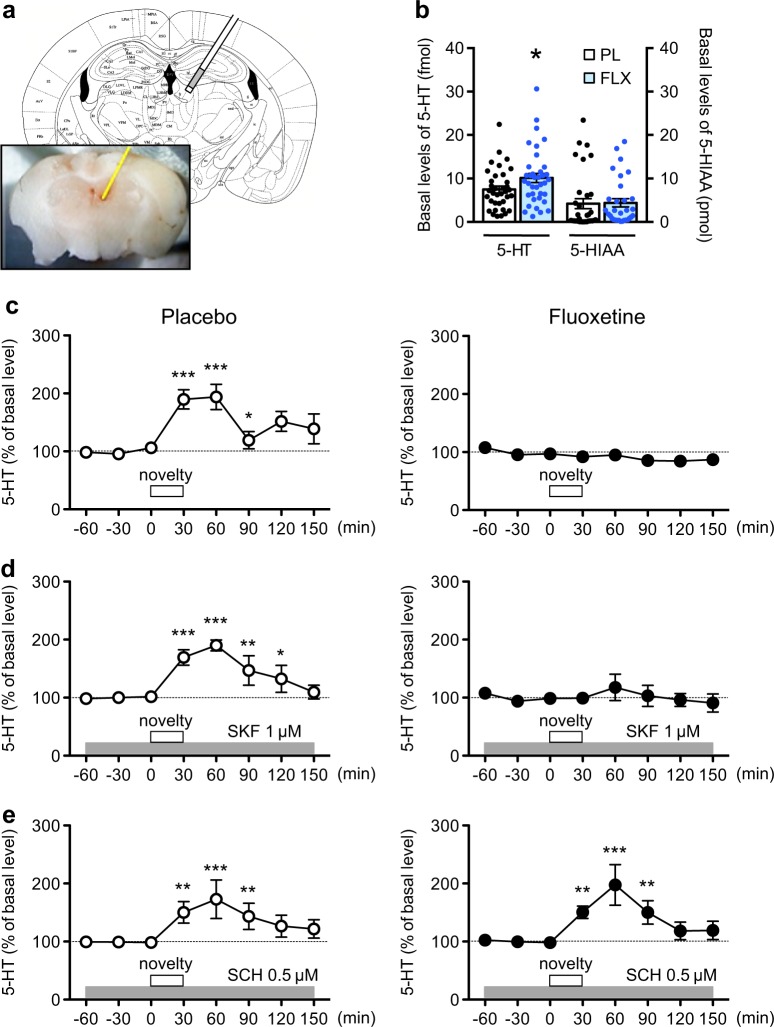
Effects of chronic fluoxetine treatment on 5-HT response to novelty stress. **a** Representative location of a microdialysis probe placed in the mouse dentate gyrus. The end of the dialysis probe is close to the habenula, but the end of the probe indicated with white color is inactive area. **b** Basal values of extracellular 5-HT and 5-HIAA contents in dialysates from the dentate gyrus of mice treated with the placebo (PL) or fluoxetine (FLX; 15 mg/kg/day for 14 days) pellet. Data are expressed as mean ± SEM. *p *<* 0.05 vs. the placebo group; Student’s *t* test. **c** Effects of novelty stress on 5-HT levels in dialysates from the dentate gyrus of mice treated with placebo (*n* = 5) or fluoxetine (*n* = 5). The open squares indicate the period (30 min) of novelty stress. **d** Effects of a local infusion of a D1 receptor agonist, R(+)-SKF81297 (1 µM), into the dentate gyrus on the 5-HT response to novelty stress in mice treated with placebo (*n* = 5) or fluoxetine (*n* = 5). **e** Effects of a local infusion of a D1 receptor antagonist, SCH23390 (0.5 µM), on the 5-HT response to novelty stress in mice treated with placebo (*n* = 5) or fluoxetine (*n* = 6). The data are expressed as mean ± SEM. *p < 0.05, **p < 0.01, ***p < 0.001 vs. the basal levels of dopamine in the same group

Analyses using in vivo microdialysis revealed that chronic fluoxetine treatment increased the basal level of 5-HT (135% of control) in the dentate gyrus (Fig. 2a, b). The increase in 5-HT level was much smaller than the acute effect of fluoxetine in the dentate gyrus (200% of control) (Supplementary Figure 6a). Basal levels of 5-hydroxyindoleacetic acid (5-HIAA), dopamine, 3,4-Dihydroxyphenylacetic acid and homovanillic acid were similar between chronic placebo and fluoxetine groups (Fig. 2b, Supplementary Figure 6b).

Upon exposure of mice to novel environment (novelty stress), a transient increase in 5-HT level was observed, up to 200% of the basal level, in placebo-treated mice (Fig. 2c). However, in fluoxetine-treated mice, the 5-HT was not increased in response to novelty stress (Fig. 2c). In contrast, novelty stress induced an increase in the dopamine level in placebo- and fluoxetine-treated mice (Supplementary Figure 6c).

We next investigated whether D1 receptors are involved in suppression of the 5-HT response in chronic fluoxetine-treated mice. Local infusion of a D1 receptor agonist, SKF81297, at 10 µM into the dentate gyrus induced a decrease in 5-HT level only in fluoxetine-treated mice (Supplementary Figure 7a). Infusion of a D1 receptor antagonist, SCH23390, at 10 µM increased 5-HT level in both groups of mice (Supplementary Figure 7b). Interestingly, SCH23390 infusion at a low concentration (0.5 µM), but not SKF81297 infusion (1 µM), reversed the suppression of the 5-HT response to novelty stress in fluoxetine-treated mice (Fig. 2d, e). Although SCH23390 is known to activate 5-HT_2C_ receptors, it is unlikely that the SCH23390 effect is mediated by 5-HT_2C_ receptors, because activation of 5-HT_2C_ receptors is expected to suppress the 5-HT response to stress [37] and 5-HT_2C_ receptors are known to be desensitized after chronic SSRI treatment [37, 38]. Taken together, these results demonstrate that chronic fluoxetine treatment upregulates D1 receptor signaling, resulting in the suppression of the 5-HT responses to novelty stress.

### Contribution of D1 receptors to fluoxetine-induced neurogenesis in the dentate gyrus

**Fig. 3:**
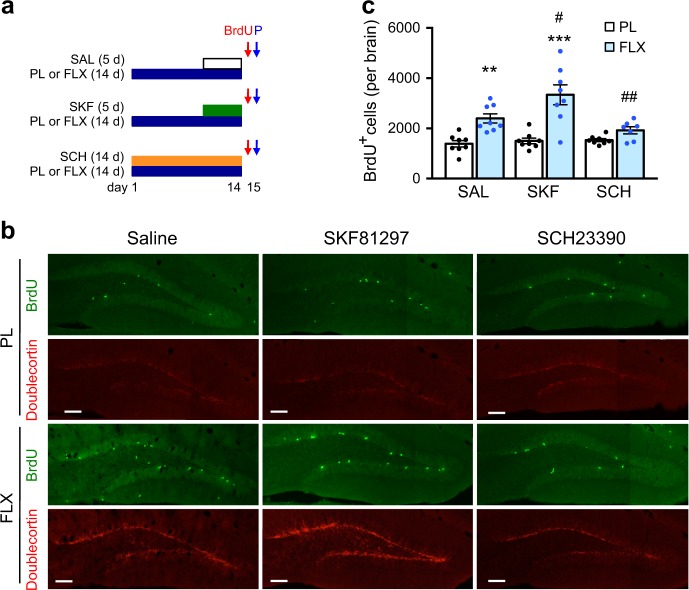
Role of fluoxetine-induced D1 receptors in adult neurogenesis in the dentate gyrus. **a** Experimental design for treatment of mice with placebo (PL) or fluoxetine (FLX; 15 mg/kg/day for 14 days) pellet and injection of R(+)-SKF81297 (SKF; 1.5 mg/kg/day i.p. for 5 days), SCH23390 (SCH; 0.1 mg/kg/day i.p. for 14 days) or saline (SAL) and for neurogenesis analysis with BrdU injection and perfusion (P). On day 15, mice were perfused 2 h after BrdU injection to evaluate cell proliferation. **b**, **c** Immunostaining with BrdU and doublecortin antibodies in the dentate gyrus (**b**) and quantitation of BrdU-positive cells in the subgranular zone of the dentate gyrus on both sides in placebo or fluoxetine pellet-treated mice (*n* = 7–8) (**c**). Scale bars, 100 µm. **p < 0.01 vs. the placebo/saline group; ***p < 0.001 vs. the placebo/SKF81297 group; two-way ANOVA and Bonferroni post hoc test. ^#^*p* < 0.05 vs. the fluoxetine/saline group; ^##^*p* < 0.01 vs. the fluoxetine/SKF81297 group; one-way ANOVA and Newman−Keuls post hoc test

Chronic fluoxetine treatment induced an increase in the number of BrdU-labeled neural progenitors compared to the placebo group (Fig. 3). Activation of D1 receptors by SKF81297 administration enhanced the effect of fluoxetine on the proliferation of neural progenitors. When D1 receptors were blocked by SCH23390 administration, fluoxetine failed to increase the proliferation of neural progenitors compared to the placebo group. Analyses along the rostrocaudal axis revealed that chronic fluoxetine treatment increases neurogenesis both in the rostral and caudal parts of the dentate gyrus, although more prominently in the caudal part (Supplementary Figure 8). The increase in neurogenesis was D1 receptor-dependent both in the rostral and caudal parts, but SKF81297 administration enhanced neurogenesis only in the caudal part. We next analyzed the expression of doublecortin, a marker of postmitotic neuroblasts. Consistently, the immunofluorescence signal of doublecortin was increased by chronic fluoxetine treatment, which was further enhanced by SKF81297 administration, but attenuated by SCH23390 administration (Fig. 3b). These findings suggest that activity of D1 receptors is required for the neurogenic effect of chronic fluoxetine treatment in the hippocampus.

### Role of D1 receptors in behavioral responses to chronic fluoxetine treatment

**Fig. 4:**
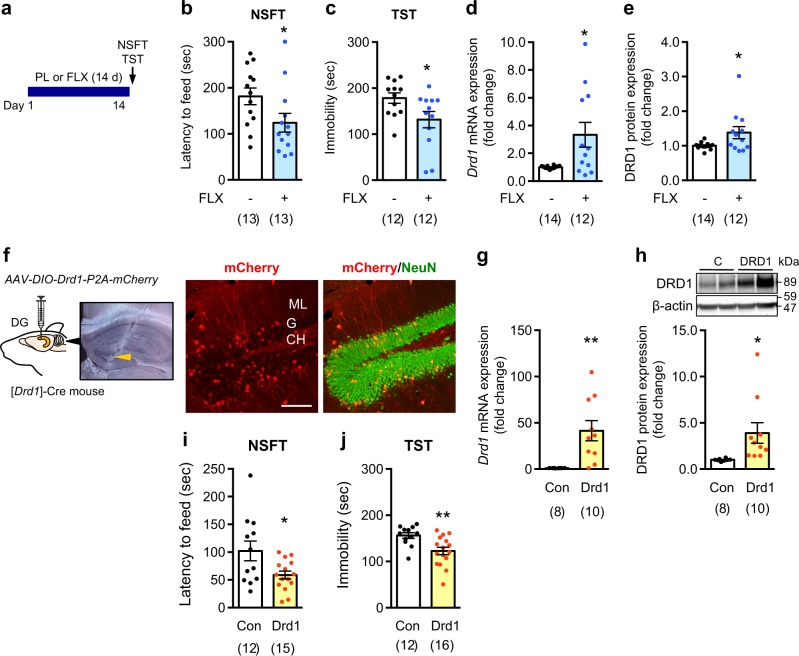
Role of D1 receptors on behavioral effects of chronic fluoxetine treatment. **a**−**e** Effects of treatment with the placebo (PL) or fluoxetine (FLX; 15 mg/kg/day for 14 days) pellet on the latency to feed in the novelty-suppressed feeding test (NSFT) (**b**) and the immobility time in the tail suspension test (TST) (**c**). After the behavioral tests, the expression levels of Drd1 mRNA (**d**) and protein (**e**) in the dentate gyrus were evaluated. *p < 0.05 vs. the placebo group; Student’s *t* test. **f**−**j** AAV vectors (*AAV-DIO-Drd1-P2A-mCherry or AAV-DIO-YFP*) were injected into both sides of the dentate gyrus of [*Drd1*]-Cre mice to overexpress D1 receptors. An image of the dentate gyrus obtained on the same day of needle insertion showed that the edge of injection needle (arrow head) was in the right place. mCherry was mainly expressed in NeuN-positive granule cells of the dentate gyrus after injection of *AAV-DIO-Drd1-P2A-mCherry* (**f**). ML molecular layer, GC granule cell layer, H hilus. Scale bars, 100 µm. Overexpression of D1 receptors in the dentate gyrus was confirmed by measurements of mRNA (**g**) and protein (**h**) levels. D1 receptor overexpression in the dentate gyrus decreased the latency to feed in the NSFT (**i**) and the immobility time in the TST (**j**). Data are expressed as mean ± SEM. *p < 0.05, **p < 0.01 vs. the control (C) group; Student’s *t* test. The number of mice is indicated in parentheses under each experimental condition

In unstressed mice, chronic fluoxetine treatment reduced the feeding latency in the NSFT and the immobility time in the TST (Fig. 4a−c). These behavioral changes were accompanied by increased levels of Drd1 mRNA and protein in the dentate gyrus (Fig. 4d, e).

To investigate the contribution of the increased expression of D1 receptors to behavioral responses, D1 receptors were overexpressed by injecting *AAV-DIO-Drd1-P2A-mCherry* into the dentate gyrus of [*Drd1*]-Cre mice. mCherry was expressed mainly in granule cells in a *Drd1 promoter* activity-dependent manner (Fig. 4f). Both mRNA and protein levels of Drd1 in the dentate gyrus were significantly increased compared to control mice (Fig. 4g, h). The overexpression of D1 receptors in granule cells reduced the feeding latency in the NSFT and the immobility time in the TST (Fig. 4i, j), without affecting the locomotor activity (control 2156 ± 203 vs. Drd1 2028 ± 236 counts/60 min). These results suggest that the elevated level of D1 receptors in granule cells is sufficient to mimic the behavioral outcomes of chronic fluoxetine treatment.

Because D1 receptors in the dentate gyrus have been implicated in seizures [39], we examined whether fluoxetine-induced expression of D1 receptors in the dentate gyrus affects epileptiform activity. Acute injection of SKF81297 at 3.0 mg/kg (i.p.), but not at 1.5 mg/kg, induced behavioral seizures of phases 2 and 3 [40] in fluoxetine-treated mice (Supplementary Figure 9), but not in placebo-treated mice. Thus, chronic fluoxetine-induced expression of D1 receptors contributes to the decrease in seizure threshold to D1 receptor activation.

### Enhancement of behavioral response to fluoxetine by D1 receptors in stressed mice

**Fig. 5:**
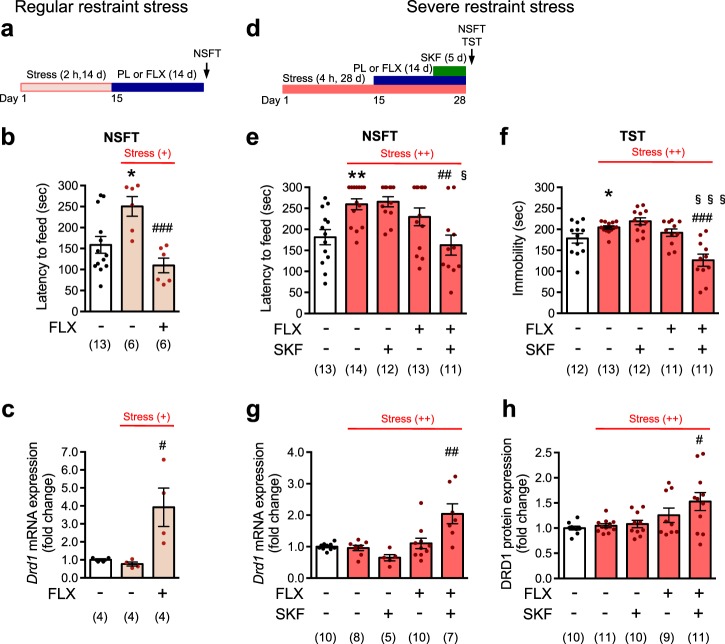
D1 receptor activation enhances antidepressant actions under severe stress conditions. **a**−**c** Regular restraint stress. **a** An experimental design for regular restraint stress and fluoxetine (FLX; 15 mg/kg/day for 14 days) administration. **b** The latency to feed in NSFT was evaluated on day 29 in mice subjected to regular restraint stress. **c** mRNA levels of *Drd1* in the dentate gyrus were evaluated after behavioral analyses. *p < 0.05 vs. the stress (−)-placebo group; ^#^*p* < 0.05, ^###^*p* < 0.001 vs. stress (+)-placebo group; Student’s *t* test. **d−h** Severe restraint stress. **d** An experimental design for severe restraint stress and fluoxetine and R(+)-SKF81297 (SKF; 1.5 mg/kg i.p. for 5 days) administration. The latency to feed in the NSFT (**e**) and the immobility time in TST (**f**) were evaluated on day 29 in mice subjected to severe restraint stress. mRNA (**g**) and protein (**h**) levels of Drd1 in the dentate gyrus were evaluated after behavioral analyses. Dissected tissues of the dentate gyrus from hippocampal slices were randomly split into mRNA and protein analyses, although all the tissues were used for either mRNA or protein analysis in some mice. *p < 0.05, **p < 0.01 vs. stress (-)-placebo/saline group; Student’s *t*-test. ^#^*p* < 0.05, ^##^*p* < 0.01, ^###^*p* < 0.001 vs. stress ( + + )-placebo/saline group; ^§^*p* < 0.05, ^§§§^*p* < 0.001 vs. stress (++)-fluoxetine/saline group; one-way ANOVA and Newman−Keuls post hoc test. The number of mice is indicated in parentheses under each experimental condition

In mice subjected to chronic restraint stress, regular restraint stress (restraint stress 2 h/day for 14 days) (Fig. 5a) increased the feeding latency in the NSFT, and the increase was reduced by chronic fluoxetine treatment (Fig. 5b). Under conditions of regular restraint stress, chronic fluoxetine treatment induced an increase in *Drd1* mRNA expression (Fig. 5c).

To investigate the role of a D1 receptor agonist, SKF81297, stress severity was increased and the stress was continued during fluoxetine treatment (severe restraint stress: 4 h/day for 28 days) (Fig. 5d), which might mimic clinical features of treatment-resistant depression: severe stress and no relief from the stress even after antidepressant medication [41]. Under conditions of severe restraint stress, the feeding latency in the NSFT was increased, but the increase was not due to locomotor deficit (Supplementary Figure 10a). Chronic fluoxetine treatment alone failed to reduce the stress-induced increase in feeding latency in the NSFT. However, chronic treatment with a D1 receptor agonist, SKF81297, together with fluoxetine, but not SKF81297 alone, reduced the increased feeding latency (Fig. 5e). In the TST, the severe restraint stress increased the immobility time, and chronic treatment of fluoxetine and SKF81297, but not either alone was able to reduce the increased immobility time (Fig. 5f).

Severe restraint stress by itself did not affect the expression of D1 receptors or markers of mature granule cells (Fig. 5g, h, Supplementary Figure 10b,c). In agreement with behavioral studies, chronic fluoxetine treatment alone failed to increase the expression levels of D1 receptors (mRNA and protein) (Fig. 5g, h). Chronic administration of SKF81297 with fluoxetine, but not SKF81297 alone, significantly increased the expression levels of D1 receptors in severely stressed mice. Under severely stressed condition, the ability of fluoxetine to reduce mRNA expression of mature granule cell markers, *Calb1* and *Tdo2*, is also restored by chronic coadministration of SKF81297 (Supplementary Figure 10b,c). Thus, together with the data for *Drd1* gene, we confirmed the requirement of D1 receptor signaling for fluoxetine-induced gene regulation under severely stressed condition: potentiation of gene expression (in case of *Drd1*) or suppression of gene expression (in case of *Calb1* and *Tdo2*). Altogether these results show tight association between the increase in D1 receptor expression and behavioral improvement and suggest a necessity of D1 receptor activity for behavioral effect of fluoxetine under severely stressed conditions.

## Discussion

This study demonstrated that chronic treatment with the antidepressant, fluoxetine, induced transcriptional activation of the *Drd1* gene in mature granule cells of the dentate gyrus. Our results suggest that D1 receptors in the dentate gyrus may improve therapeutic efficacy of antidepressants via multiple mechanisms (Supplementary Figure 11). Chronic fluoxetine-induced D1 receptors were functionally coupled with adenylyl cyclase, leading to upregulation of cAMP/PKA signaling and thereby increased neuronal excitability [13]. D1 receptors also play a critical role in fluoxetine-induced attenuation of the 5-HT response to novelty stress and fluoxetine-induced adult neurogenesis in the SGZ of the dentate gyrus. An increase of D1 receptor expression in the dentate gyrus by chronic fluoxetine treatment was tightly correlated with improved behavioral outcome. In addition, overexpression of D1 receptors in the dentate gyrus was sufficient to induce antidepressant-like behavioral effects. Under severely stressed conditions, chronic fluoxetine treatment alone failed to induce the expression of D1 receptors and improve depression-like behaviors. However, coadministration of the D1 receptor agonist enhanced the action of fluoxetine, leading to the induction of D1 receptors and the improvement of depression-like behaviors.

### Induction of D1 receptors by antidepressants in granule cells of the dentate gyrus

D1 receptors are expressed in a subset of granule cells and GABAergic interneurons such as pyramidal basket cells, axo-axonic cells and molecular perforant path-associated cells in the dentate gyrus [32, 42]. Chronic fluoxetine administration robustly induced the expression of *Drd1 promoter*-driven EGFP in a large majority of NeuN-positive granule cells throughout the dentate gyrus of [*Drd1*]-EGFP mice. The results are in agreement with previous reports showing the fluoxetine-induced increase in Drd1 mRNA in the hippocampus [43] and [^3^H]SCH23390 binding to D1 receptors in the dentate gyrus [12].

Chronic fluoxetine treatment increases adult neurogenesis by 40–60% in the dentate gyrus [22, 44], and induces activation of the *Drd1 promoter* in doublecortin-positive immature neurons at the SGZ (Supplementary Figure 2b). However, newborn granule cells (within 4 weeks after differentiation) are estimated to constitute less than 6% of the total granule cells in the dentate gyrus [45], and do not replace existing, mature granule cells [46]. Therefore, contribution of newborn granule cells to the increased D1 receptor expression in the dentate gyrus is fractional.

The mechanism by which chronic fluoxetine treatment induces D1 receptor expression in mature granule cells is not known. The induction of D1 receptors is likely initiated through 5-HT neurotransmission via 5-HT4 receptors in granule cells [13], and potentiated through D1 receptor signaling presumably via the phosphorylation of PKA substrates including phosphorylation of DARPP-32 at Thr34. Notably, we also observed a decrease of phosphorylation level at Ser97 on DARPP-32 after chronic fluoxetine treatment (Supplementary Figure 5b). Dephosphorylation at Ser97 of DARPP-32 is associated with nuclear translocation of DARPP-32 and its involvement in epigenetic regulation and gene transcription [35]. Thus, chronic fluoxetine treatment likely causes accumulation of nuclear DARPP-32 having phospho-Thr34/dephospho-Ser97, which in turn inhibits nuclear protein phosphatase 1, resulting in an increase of phosphorylation of histone H3 and transcriptional activation. Histone H4 acetylation of the *Drd1 promotor region* has been implicated in nicotine-induced *Drd1* mRNA expression in the prefrontal cortex [47], supporting a possible involvement of an epigenetic regulatory mechanism in chronic fluoxetine-induced Drd1 induction.

Transcriptional activation of the *Drd1* gene in granule cells is also observed in electroconvulsive treatment of mice [48], pilocarpine-induced seizures [49] and α-CaMKII+/− mice [50]. In α-CaMKII+/− mice, granule cells are likely arrested at a pseudoimmature state in molecular and electrophysiological properties and show hyperexcitability, and the phenomenon is referred to as the immature dentate gyrus [50, 51]. These studies suggest that the increased excitability of granule cells may be a critical factor to activate transcription of the *Drd1* gene. In addition, it has been reported that the *Drd1 promotor* region has binding sites for transcription factors such as Sp1, Ap1, and Ap2 and potential cAMP and glucocorticoid response element sequences [52, 53], and that the *Drd1 promotor* is positively regulated by cAMP [53]. Further studies are required to elucidate the precise mechanisms of chronic fluoxetine-induced *Drd1* gene transcription in the dentate gyrus.

Chronic fluoxetine treatment failed to increase D1 receptor expression in the dentate gyrus in mice subjected to severe restraint stress. Stress has been shown to induce the alteration of gene expression profiles depending on the stress-type [54]. The increase or decrease of gene expression is mediated through multiple mechanisms including activity of the hypothalamic-pituitary-adrenal (HPA) axis and the levels of glucocorticoid [55], transcription factors that regulate *Drd1 promoter* activity [56], and epigenetic mechanisms [57]. Severe restraint stress may induce the alteration in gene expression profile and epigenetic modification of the *Drd1 promoter*, which suppresses the pathways of fluoxetine-induced D1 receptor expression.

### Functional implication of upregulated D1 receptor signaling in hippocampal neural circuits

It has been shown that chronic fluoxetine treatment increases the excitability of granule cells [13], and that D1 receptors enhance LTP at the mossy fiber-CA3 pyramidal neuron synapse [12, 58] and perforant path-granule cell synapses [42, 59]. Upregulation of D1 receptors by chronic fluoxetine treatment likely contributes to the enhancement of LTP. In addition to DARPP-32 phosphorylation, we also demonstrated increased phosphorylation of GluA1 and ERK as a result of upregulation of D1 receptor/cAMP/PKA signaling (Supplementary Figure 5c and d). The potentiation of these downstream pathways may contribute to the increase in granule cell’s excitability and synaptic transmission, resulting in stimulation of hippocampal circuits throughout the dentate gyrus.

### Role of D1 receptors in 5-HT release in the dentate gyrus in response to novel environment-evoked stress

During exposure to psychological and nociceptive stressors, the levels of 5-HT in the hippocampus are known to increase [60, 61]. It has been known that acute exposure to SSRIs exacerbates symptoms of anxiety in patients with anxiety disorders [62]. Studies in rodents also demonstrated that acute administration of SSRIs resulting in a rapid increase in extracellular 5-HT levels induces anxiogenic effects, whereas chronic administration of SSRIs induces anxiolytic effects [63, 64]. Recently, activation of the serotonergic circuit from the dorsal raphe nucleus to the bed nucleus of the stria terminalis was shown to enhance anxiety and fear [65]. These observations suggest that the increase in 5-HT levels in response to acute stress may be associated with negative mood including anxiety. In this study, chronic fluoxetine treatment attenuates the novel environment-induced 5-HT release in the dentate gyrus. The outcome of our study suggests that this attenuation of the 5-HT response is mediated by upregulated D1 receptor signaling after chronic fluoxetine treatment.

Despite the significant role of D1 receptor induction, the precise mechanism by which the D1 receptor in the dentate gyrus regulates 5-HT neurotransmission is currently unknown. Activation of D1 receptor signaling in mature granule cells increases their excitability [12, 42, 58], which may further propagate to their postsynaptic targets including GABAergic interneurons and mossy cells in the dentate gyrus, and pyramidal neurons in CA subfields through mossy fibers [66]. The increased GABAergic tone may result in the suppression of 5-HT release from serotonergic terminals [67]. Furthermore, hyperexcitability of dentate granule cells may strengthen multiple extrahippocampal projections from CA3 and CA1 pyramidal neurons to the hypothalamus, amygdala and prefrontal cortex [68], which may contribute to indirect circuitry to regulate the activity of serotonergic neurons in the dorsal raphe nucleus [69]. It is unlikely that D1 receptors expressed at serotonergic terminals directly induce the attenuation of the 5-HT response, because activation of D1 receptors at serotonergic terminals may induce the enhancement, instead of the attenuation, of the 5-HT response [70, 71]. In a future study, it would be important to identify the exact circuitry mechanisms to explain the D1 receptor-mediated attenuation of the 5-HT response to novel environment.

### Role of D1 receptor induction in antidepressant-induced neurogenesis

Neurogenesis in the SGZ of the dentate gyrus is downregulated under stressful conditions and depressive states, and upregulated by antidepressant treatment [9, 10, 22, 44]. The immature newborn neurons are integrated to a local dentate circuit, and modulate the function of mature granule cells and hippocampal circuits [46]. Studies in dopamine-depleted rodent models of Parkinson’s disease have implicated a role for dopamine in adult neurogenesis in the SGZ [72]. Neural precursors in the SGZ are innervated by dopaminergic afferents [72], and seem to express all subtypes of dopamine receptors [73]. In experiments with naïve or dopamine-depleted rodents, D1 receptors were shown to promote the survival of newborn cells [74], whereas D2 receptors were shown to promote the proliferation of neural precursors [72, 75]. When neurogenesis was stimulated with chronic fluoxetine treatment, we found that D1 receptors showed the ability to promote the proliferation of neural precursors in the SGZ of both rostral and caudal dentate gyrus, but more prominently in the caudal dentate gyrus. It is possible that D1 receptors in neural precursors play a role in regulating the proliferation autonomously, although the D1 receptor expression barely overlaps with a proliferating cell marker (Ki67) or an immature neuron maker (calretinin) in the SGZ in this study. The D1 receptor expression in doublecortin-positive neuroblasts suggests the involvement of D1 receptors in immature neurons for their survival and differentiation. In addition, D1 receptor expression in mature granule cells and the subsequent increase in neural activity are likely to contribute to antidepressant-induced neurogenesis via non-cell autonomous mechanisms [76].

### The D1 receptor as a potential therapeutic target for antidepressant-resistant depression

The hypodopaminergic state has been implicated in the pathophysiology of depression [11]. Previous studies implicated a potential role for D1 receptors in the chronic actions of antidepressants. For example, a D1 receptor antagonist reversed chronic antidepressant effects of tricyclic antidepressants on learned helplessness acquisition [15]. However, the brain region in which D1 receptors promote the chronic actions of antidepressants has not been identified. In the present study, we show that pharmacological activation of D1 receptors together with chronic fluoxetine treatment induces the expression of D1 receptors in the dentate gyrus and improved severe restraint stress-induced depression-like behaviors, which were resistant to chronic fluoxetine treatment. The results raise the possibility that the D1 receptor may be considered as a therapeutic target for treatment-resistant depression. As activation of D1 receptors alone does not improve depression-like behaviors, the D1 receptor agonist may not be used as a monotherapy. However, when combined with chronic antidepressant treatment, the D1 receptor agonist is likely to potentiate therapeutic effects. In clinical studies, the Sequenced Treatment Alternatives to Relieve Depression (STAR*D) trial demonstrated that bupropion, a dopamine and noradrenaline reuptake inhibitor, augments antidepressant action of SSRI in patients with incomplete response to initial SSRI treatment [77, 78], supporting the role of D1 receptors for the enhancement of antidepressant action.

A therapeutic approach to increase D1 receptor signaling requires improvement of D1 agonists to overcome drawbacks such as low oral bioavailability and short half-life [79]. Safety concerns about seizures and hypotension also need to be dealt with [80, 81]. It has been hypothesized that therapeutic efficacy of antidepressants coincides with the inverted U shape D1 receptor activation-response in the PFC [11], as has been demonstrated for the role of D1 receptors in the PFC in cognitive function [79]. Interestingly, changes in the expression-states of D1 receptors from overexpression to relatively reduced expression in the PFC are closely associated with a switch from mania/hedonia to depression/anhedonia [82]. These functional features of D1 receptors in the PFC are somewhat similar to the features of D1 receptors in the dentate gyrus, where upregulation of D1 receptor signaling exerts therapeutic effects in the depressive state, but its overactivation results in seizure induction and a possible shift to the manic state. Development of improved D1 receptor agonists for clinical practice and of monitoring methods for optimal activation of D1 receptors are necessary for a therapeutic approach using D1 receptor agonists for treatment-resistant depression.

## Supplementary information

Supplementary Figure 1

Supplementary Figure 2

Supplementary Figure 3

Supplementary Figure 4

Supplementary Figure 5

Supplementary Figure 6

Supplementary Figure 7

Supplementary Figure 8

Supplementary Figure 9

Supplementary Figure 10

Supplementary Figure 11

Supplementary Table 1

Supplementary Table 2

Supplementary Table 3
