# Supplementary material for: Obligatory roles of dopamine D1 receptors in the dentate gyrus in antidepressant actions of a selective serotonin reuptake inhibitor, fluoxetine
Source: Mol Psychiatry. 2018 Dec 10;25(6):1229–44. doi: 10.1038/s41380-018-0316-x (PMC7244404; doi:10.1038/s41380-018-0316-x)
Supplement: Supplementary file 1 — Supplementary Figure 1 [file 41380_2018_316_MOESM1_ESM.pptx]

## Slide 1
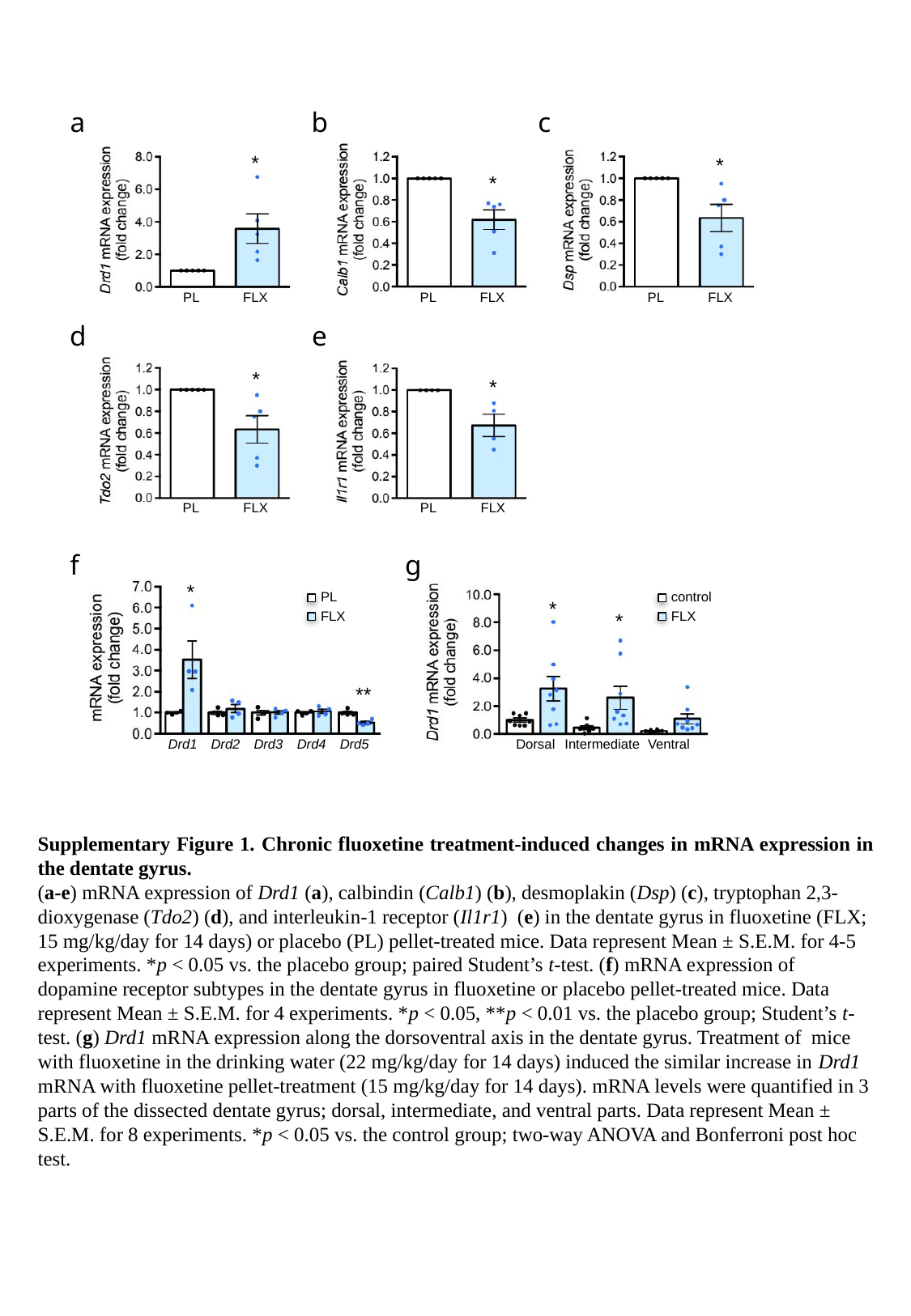

a
b
c
*
*
*
PL
FLX
PL
FLX
PL
FLX
d
e
*
*
PL
FLX
PL
FLX
f
g
*
control
PL
*
FLX
FLX
*
**
Drd1
Drd2
Drd3
Drd4
Drd5
Dorsal
Intermediate
Ventral
Supplementary Figure 1. Chronic fluoxetine treatment-induced changes in mRNA expression in the dentate gyrus.
(a-e) mRNA expression of Drd1 (a), calbindin (Calb1) (b), desmoplakin (Dsp) (c), tryptophan 2,3-dioxygenase (Tdo2) (d), and interleukin-1 receptor (Il1r1) (e) in the dentate gyrus in fluoxetine (FLX; 15 mg/kg/day for 14 days) or placebo (PL) pellet-treated mice. Data represent Mean ± S.E.M. for 4-5 experiments. *p < 0.05 vs. the placebo group; paired Student’s t-test. (f) mRNA expression of dopamine receptor subtypes in the dentate gyrus in fluoxetine or placebo pellet-treated mice. Data represent Mean ± S.E.M. for 4 experiments. *p < 0.05, **p < 0.01 vs. the placebo group; Student’s t-test. (g) Drd1 mRNA expression along the dorsoventral axis in the dentate gyrus. Treatment of mice with fluoxetine in the drinking water (22 mg/kg/day for 14 days) induced the similar increase in Drd1 mRNA with fluoxetine pellet-treatment (15 mg/kg/day for 14 days). mRNA levels were quantified in 3 parts of the dissected dentate gyrus; dorsal, intermediate, and ventral parts. Data represent Mean ± S.E.M. for 8 experiments. *p < 0.05 vs. the control group; two-way ANOVA and Bonferroni post hoc test.
