# Supplementary material for: Obligatory roles of dopamine D1 receptors in the dentate gyrus in antidepressant actions of a selective serotonin reuptake inhibitor, fluoxetine
Source: Mol Psychiatry. 2018 Dec 10;25(6):1229–44. doi: 10.1038/s41380-018-0316-x (PMC7244404; doi:10.1038/s41380-018-0316-x)
Supplement: Supplementary file 2 — Supplementary Figure 2 [file 41380_2018_316_MOESM2_ESM.pptx]

## Slide 1
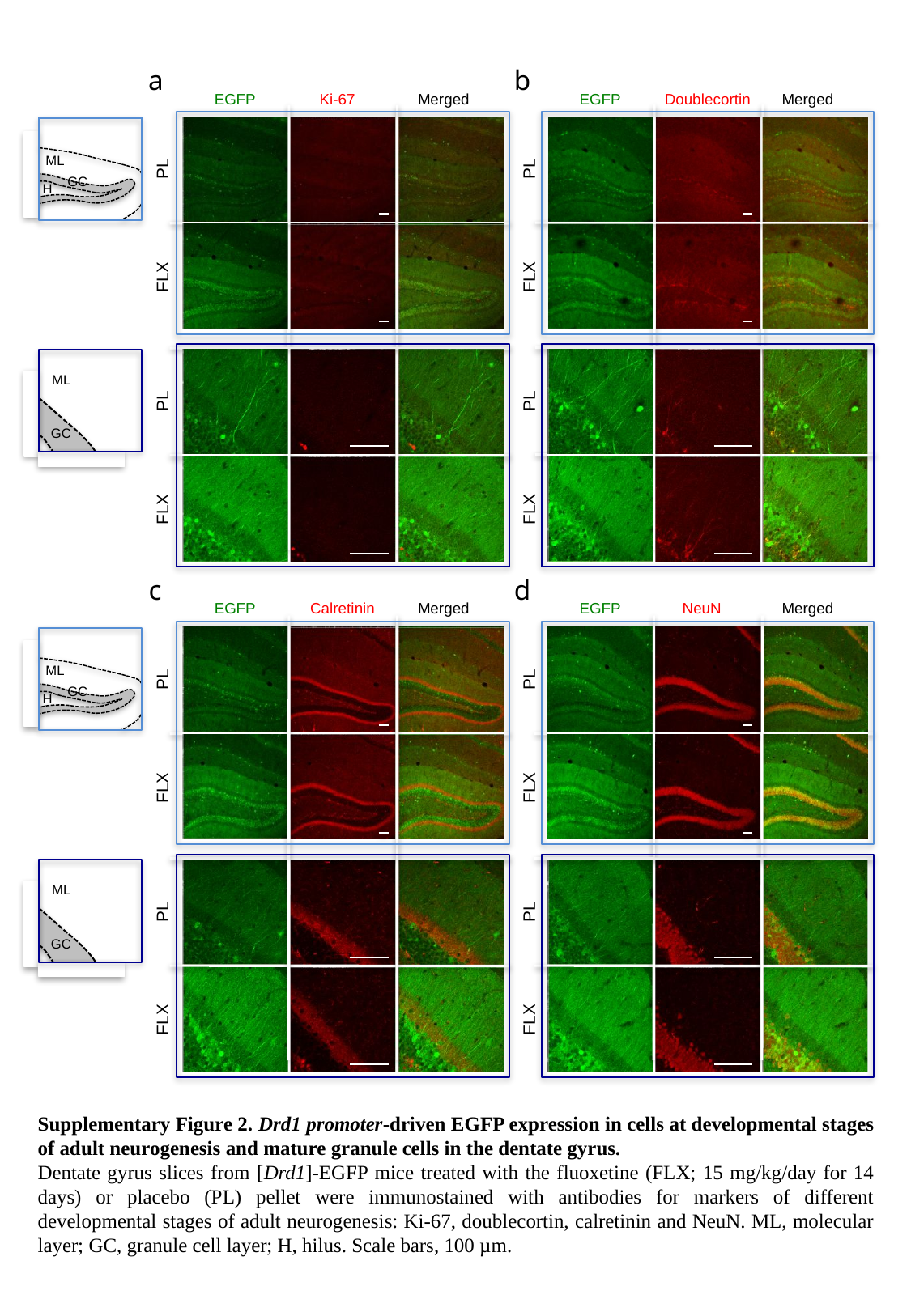

a
b
EGFP
Ki-67
Merged
EGFP
Doublecortin
Merged
ML
PL
PL
GC
H
FLX
FLX
ML
PL
PL
GC
FLX
FLX
c
d
EGFP
Calretinin
Merged
EGFP
NeuN
Merged
ML
PL
PL
GC
H
FLX
FLX
ML
PL
PL
GC
FLX
FLX
Supplementary Figure 2. Drd1 promoter-driven EGFP expression in cells at developmental stages of adult neurogenesis and mature granule cells in the dentate gyrus.
Dentate gyrus slices from [Drd1]-EGFP mice treated with the fluoxetine (FLX; 15 mg/kg/day for 14 days) or placebo (PL) pellet were immunostained with antibodies for markers of different developmental stages of adult neurogenesis: Ki-67, doublecortin, calretinin and NeuN. ML, molecular layer; GC, granule cell layer; H, hilus. Scale bars, 100 µm.
