# Supplementary material for: Obligatory roles of dopamine D1 receptors in the dentate gyrus in antidepressant actions of a selective serotonin reuptake inhibitor, fluoxetine
Source: Mol Psychiatry. 2018 Dec 10;25(6):1229–44. doi: 10.1038/s41380-018-0316-x (PMC7244404; doi:10.1038/s41380-018-0316-x)
Supplement: Supplementary file 3 — Supplementary Figure 3 [file 41380_2018_316_MOESM3_ESM.pptx]

## Slide 1
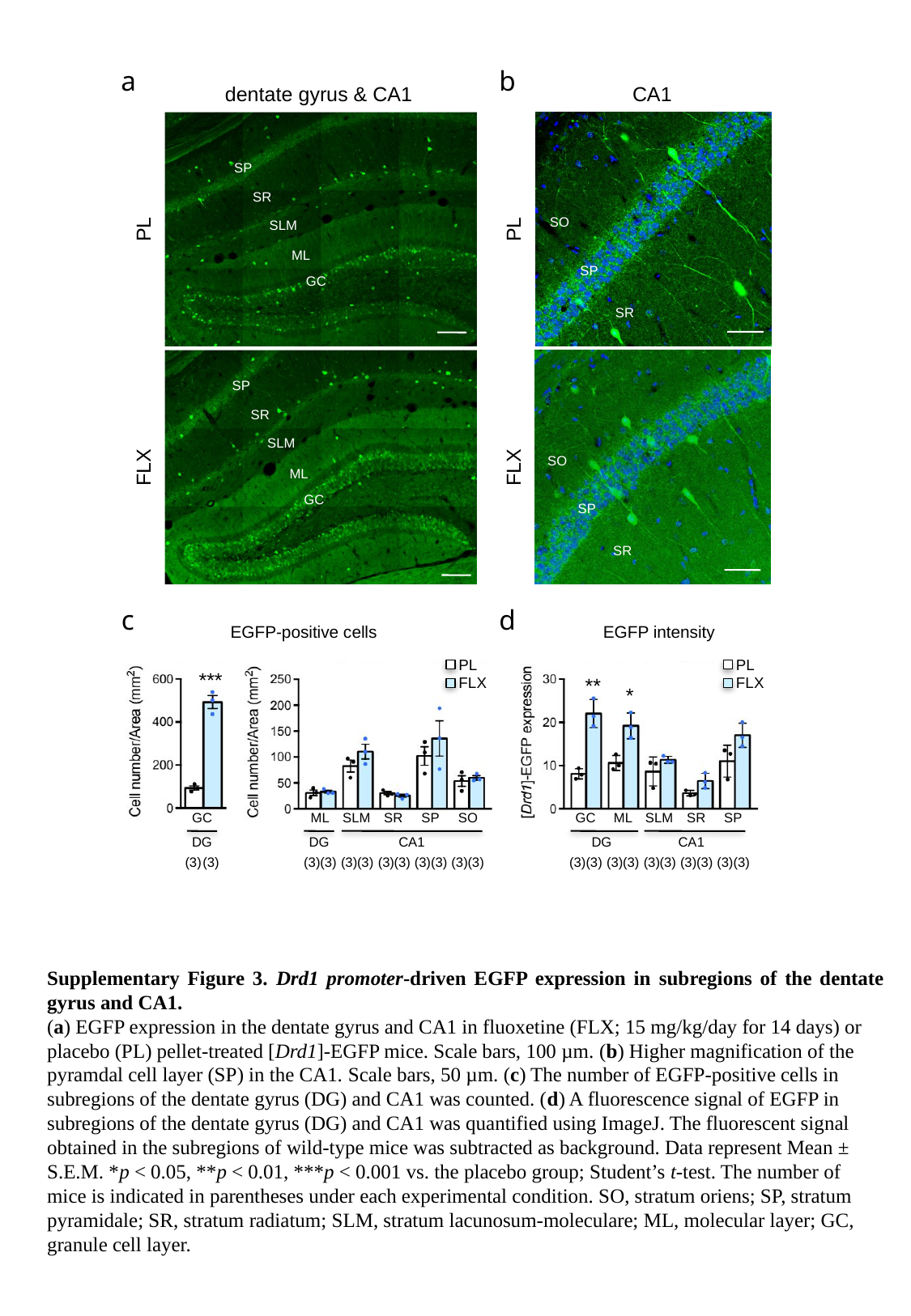

a
b
dentate gyrus & CA1
CA1
SO
SP
SR
SP
SR
PL
PL
SLM
ML
GC
SO
SP
SR
SP
SR
SLM
FLX
FLX
ML
GC
c
d
EGFP-positive cells
EGFP intensity
PL
FLX
PL
FLX
***
**
*
GC
ML
SLM
SR
SP
SO
GC
ML
SLM
SR
SP
DG
DG
CA1
DG
CA1
(3)
(3)
(3)
(3)
(3)
(3)
(3)
(3)
(3)
(3)
(3)
(3)
(3)
(3)
(3)
(3)
(3)
(3)
(3)
(3)
(3)
(3)
Supplementary Figure 3. Drd1 promoter-driven EGFP expression in subregions of the dentate gyrus and CA1.
(a) EGFP expression in the dentate gyrus and CA1 in fluoxetine (FLX; 15 mg/kg/day for 14 days) or placebo (PL) pellet-treated [Drd1]-EGFP mice. Scale bars, 100 µm. (b) Higher magnification of the pyramdal cell layer (SP) in the CA1. Scale bars, 50 µm. (c) The number of EGFP-positive cells in subregions of the dentate gyrus (DG) and CA1 was counted. (d) A fluorescence signal of EGFP in subregions of the dentate gyrus (DG) and CA1 was quantified using ImageJ. The fluorescent signal obtained in the subregions of wild-type mice was subtracted as background. Data represent Mean ± S.E.M. *p < 0.05, **p < 0.01, ***p < 0.001 vs. the placebo group; Student’s t-test. The number of mice is indicated in parentheses under each experimental condition. SO, stratum oriens; SP, stratum pyramidale; SR, stratum radiatum; SLM, stratum lacunosum-moleculare; ML, molecular layer; GC, granule cell layer.
