# Supplementary material for: Obligatory roles of dopamine D1 receptors in the dentate gyrus in antidepressant actions of a selective serotonin reuptake inhibitor, fluoxetine
Source: Mol Psychiatry. 2018 Dec 10;25(6):1229–44. doi: 10.1038/s41380-018-0316-x (PMC7244404; doi:10.1038/s41380-018-0316-x)
Supplement: Supplementary file 4 — Supplementary Figure 4 [file 41380_2018_316_MOESM4_ESM.pptx]

## Slide 1
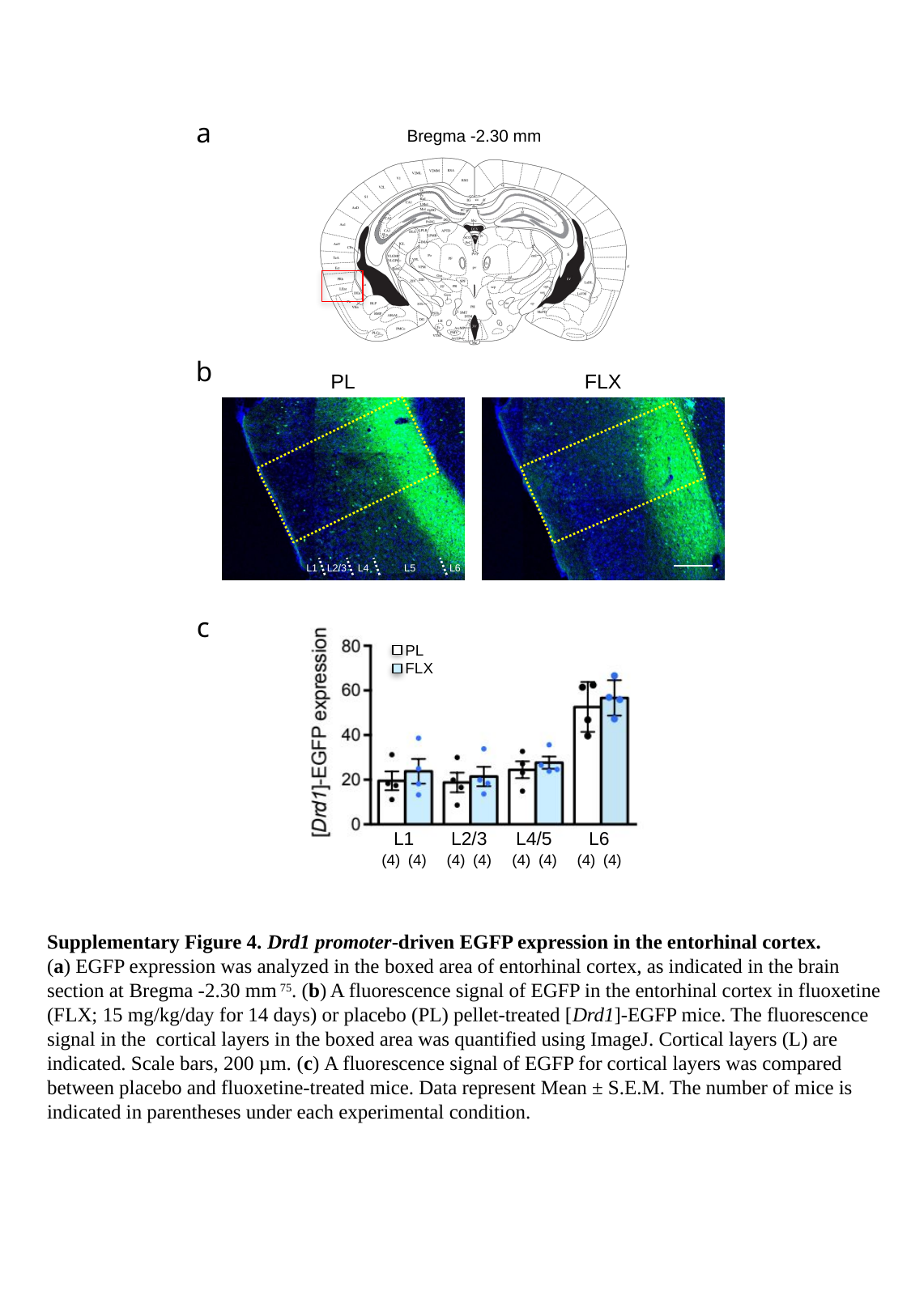

a
Bregma -2.30 mm
b
PL
FLX
L1
L2/3
L4
L5
L6
c
PL
FLX
L1
L2/3
L4/5
L6
(4)
(4)
(4)
(4)
(4)
(4)
(4)
(4)
Supplementary Figure 4. Drd1 promoter-driven EGFP expression in the entorhinal cortex.
(a) EGFP expression was analyzed in the boxed area of entorhinal cortex, as indicated in the brain section at Bregma -2.30 mm 75. (b) A fluorescence signal of EGFP in the entorhinal cortex in fluoxetine (FLX; 15 mg/kg/day for 14 days) or placebo (PL) pellet-treated [Drd1]-EGFP mice. The fluorescence signal in the cortical layers in the boxed area was quantified using ImageJ. Cortical layers (L) are indicated. Scale bars, 200 µm. (c) A fluorescence signal of EGFP for cortical layers was compared between placebo and fluoxetine-treated mice. Data represent Mean ± S.E.M. The number of mice is indicated in parentheses under each experimental condition.
