# Supplementary material for: Obligatory roles of dopamine D1 receptors in the dentate gyrus in antidepressant actions of a selective serotonin reuptake inhibitor, fluoxetine
Source: Mol Psychiatry. 2018 Dec 10;25(6):1229–44. doi: 10.1038/s41380-018-0316-x (PMC7244404; doi:10.1038/s41380-018-0316-x)
Supplement: Supplementary file 5 — Supplementary Figure 5 [file 41380_2018_316_MOESM5_ESM.pptx]

## Slide 1
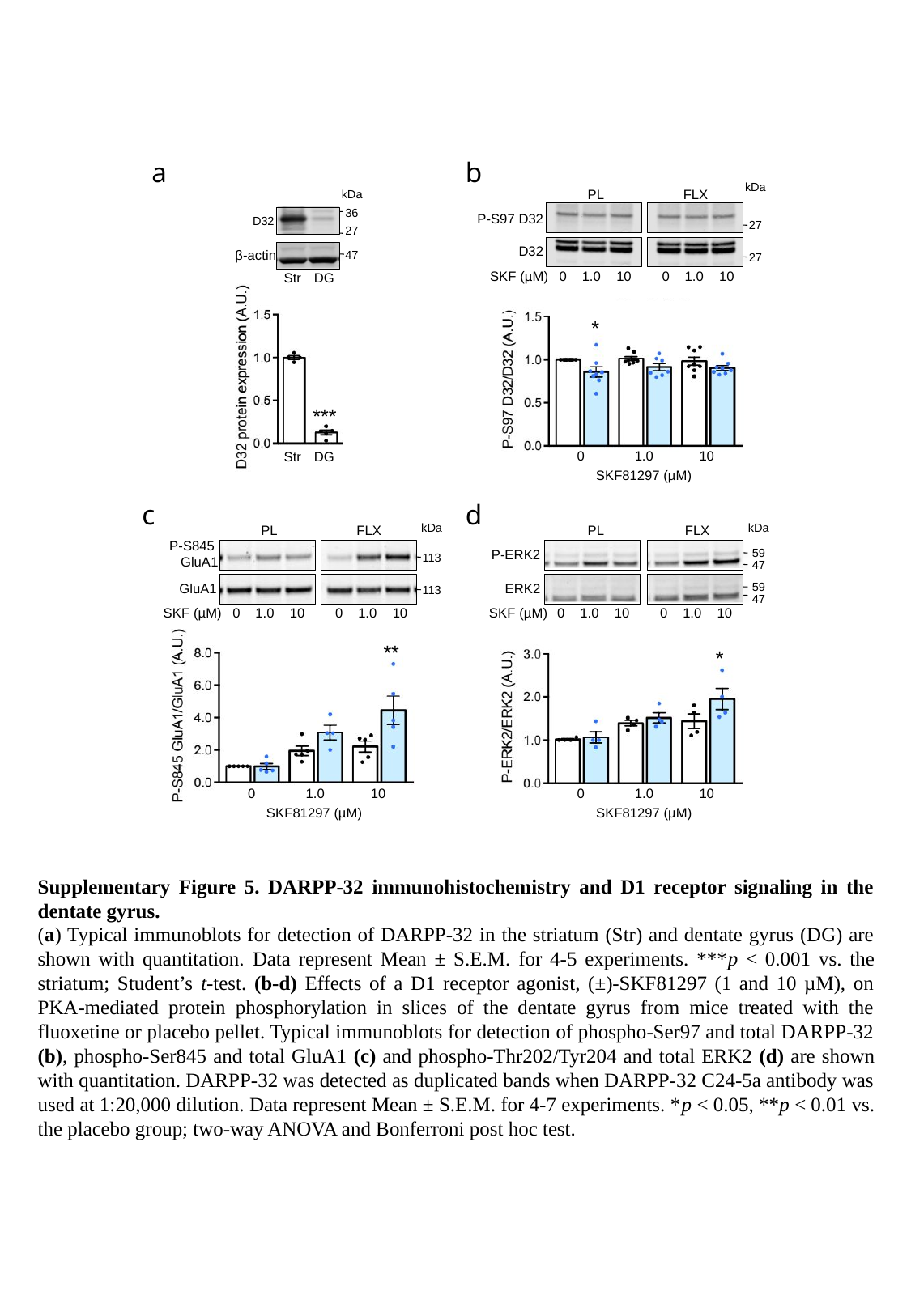

a
b
kDa
PL
FLX
P-S97 D32
27
D32
27
0
1.0
10
0
1.0
10
SKF (µM)
*
0
1.0
10
SKF81297 (µM)
kDa
36
D32
27
β-actin
47
Str
DG
***
Str
DG
c
d
kDa
PL
FLX
P-S845
GluA1
113
GluA1
113
0
1.0
10
0
1.0
10
SKF (µM)
**
0
1.0
10
SKF81297 (µM)
kDa
PL
FLX
59
P-ERK2
47
59
ERK2
47
0
1.0
10
0
1.0
10
SKF (µM)
*
0
1.0
10
SKF81297 (µM)
Supplementary Figure 5. DARPP-32 immunohistochemistry and D1 receptor signaling in the dentate gyrus.
(a) Typical immunoblots for detection of DARPP-32 in the striatum (Str) and dentate gyrus (DG) are shown with quantitation. Data represent Mean ± S.E.M. for 4-5 experiments. ***p < 0.001 vs. the striatum; Student’s t-test. (b-d) Effects of a D1 receptor agonist, (±)-SKF81297 (1 and 10 µM), on PKA-mediated protein phosphorylation in slices of the dentate gyrus from mice treated with the fluoxetine or placebo pellet. Typical immunoblots for detection of phospho-Ser97 and total DARPP-32 (b), phospho-Ser845 and total GluA1 (c) and phospho-Thr202/Tyr204 and total ERK2 (d) are shown with quantitation. DARPP-32 was detected as duplicated bands when DARPP-32 C24-5a antibody was used at 1:20,000 dilution. Data represent Mean ± S.E.M. for 4-7 experiments. *p < 0.05, **p < 0.01 vs. the placebo group; two-way ANOVA and Bonferroni post hoc test.
