# Supplementary material for: Obligatory roles of dopamine D1 receptors in the dentate gyrus in antidepressant actions of a selective serotonin reuptake inhibitor, fluoxetine
Source: Mol Psychiatry. 2018 Dec 10;25(6):1229–44. doi: 10.1038/s41380-018-0316-x (PMC7244404; doi:10.1038/s41380-018-0316-x)
Supplement: Supplementary file 6 — Supplementary Figure 6 [file 41380_2018_316_MOESM6_ESM.pptx]

## Slide 1
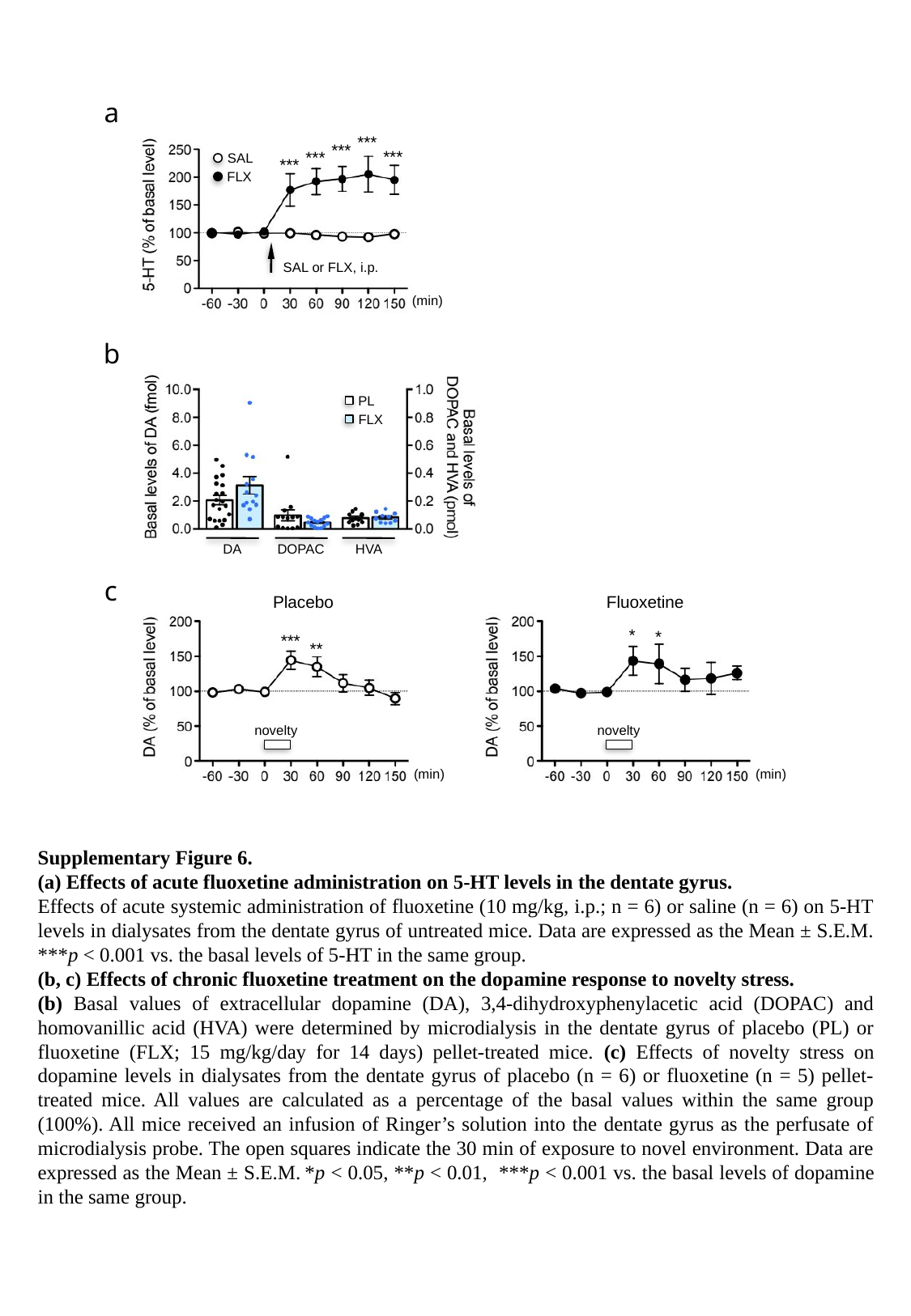

a
***
***
***
***
SAL
***
FLX
SAL or FLX, i.p.
(min)
b
PL
FLX
DA
DOPAC
HVA
c
Placebo
Fluoxetine
*
*
***
**
novelty
novelty
(min)
(min)
Supplementary Figure 6.
(a) Effects of acute fluoxetine administration on 5-HT levels in the dentate gyrus.
Effects of acute systemic administration of fluoxetine (10 mg/kg, i.p.; n = 6) or saline (n = 6) on 5-HT levels in dialysates from the dentate gyrus of untreated mice. Data are expressed as the Mean ± S.E.M. ***p < 0.001 vs. the basal levels of 5-HT in the same group.
(b, c) Effects of chronic fluoxetine treatment on the dopamine response to novelty stress.
(b) Basal values of extracellular dopamine (DA), 3,4-dihydroxyphenylacetic acid (DOPAC) and homovanillic acid (HVA) were determined by microdialysis in the dentate gyrus of placebo (PL) or fluoxetine (FLX; 15 mg/kg/day for 14 days) pellet-treated mice. (c) Effects of novelty stress on dopamine levels in dialysates from the dentate gyrus of placebo (n = 6) or fluoxetine (n = 5) pellet-treated mice. All values are calculated as a percentage of the basal values within the same group (100%). All mice received an infusion of Ringer’s solution into the dentate gyrus as the perfusate of microdialysis probe. The open squares indicate the 30 min of exposure to novel environment. Data are expressed as the Mean ± S.E.M. *p < 0.05, **p < 0.01, ***p < 0.001 vs. the basal levels of dopamine in the same group.
