# Supplementary material for: Obligatory roles of dopamine D1 receptors in the dentate gyrus in antidepressant actions of a selective serotonin reuptake inhibitor, fluoxetine
Source: Mol Psychiatry. 2018 Dec 10;25(6):1229–44. doi: 10.1038/s41380-018-0316-x (PMC7244404; doi:10.1038/s41380-018-0316-x)
Supplement: Supplementary file 7 — Supplementary Figure 7 [file 41380_2018_316_MOESM7_ESM.pptx]

## Slide 1
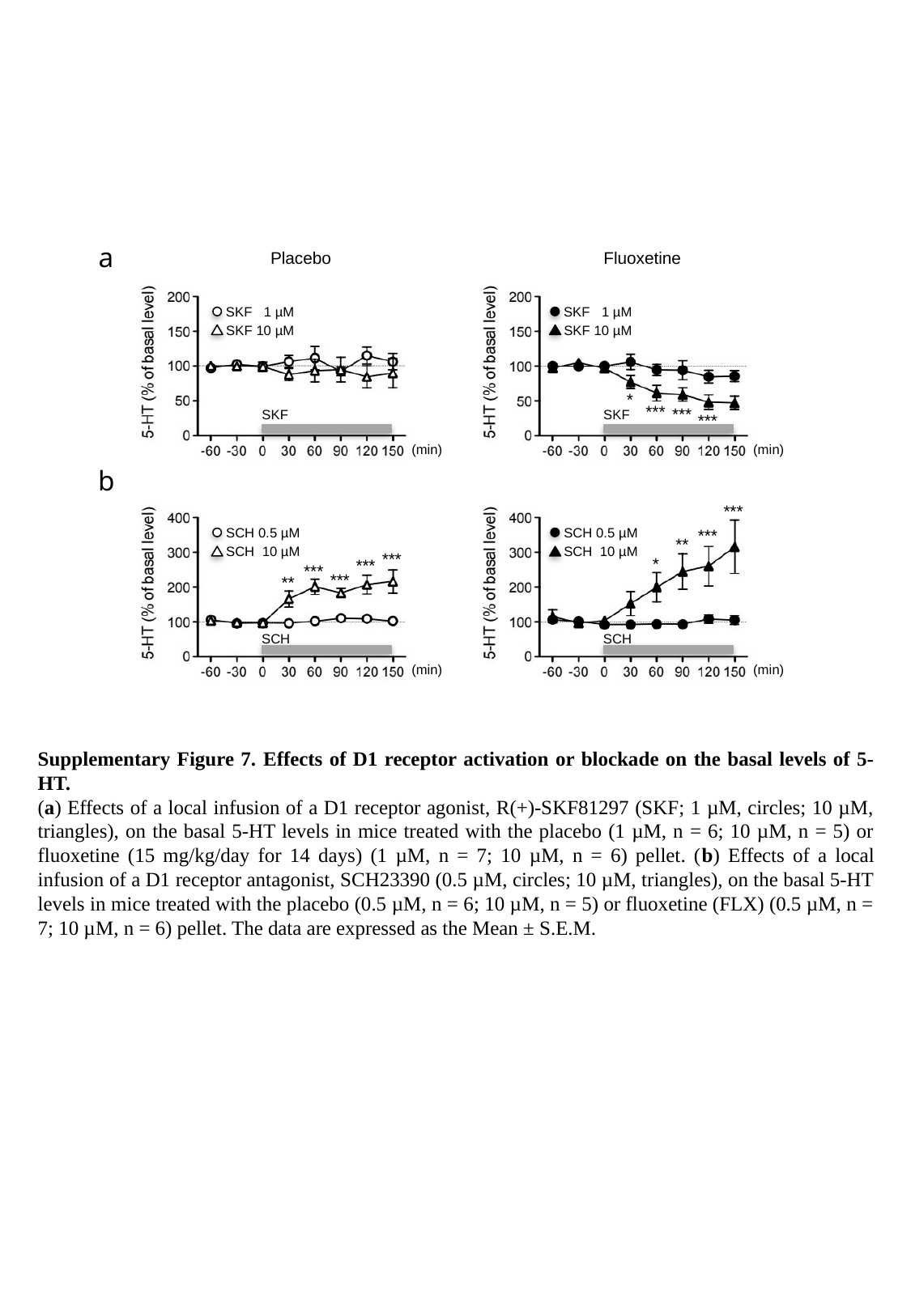

a
Placebo
Fluoxetine
SKF 1 µM
SKF 1 µM
SKF 10 µM
SKF 10 µM
*
***
***
SKF
SKF
***
(min)
(min)
b
***
SCH 0.5 µM
SCH 0.5 µM
***
**
SCH 10 µM
SCH 10 µM
***
*
***
***
***
**
SCH
SCH
(min)
(min)
Supplementary Figure 7. Effects of D1 receptor activation or blockade on the basal levels of 5-HT.
(a) Effects of a local infusion of a D1 receptor agonist, R(+)-SKF81297 (SKF; 1 µM, circles; 10 µM, triangles), on the basal 5-HT levels in mice treated with the placebo (1 µM, n = 6; 10 µM, n = 5) or fluoxetine (15 mg/kg/day for 14 days) (1 µM, n = 7; 10 µM, n = 6) pellet. (b) Effects of a local infusion of a D1 receptor antagonist, SCH23390 (0.5 µM, circles; 10 µM, triangles), on the basal 5-HT levels in mice treated with the placebo (0.5 µM, n = 6; 10 µM, n = 5) or fluoxetine (FLX) (0.5 µM, n = 7; 10 µM, n = 6) pellet. The data are expressed as the Mean ± S.E.M.
