# Supplementary material for: Obligatory roles of dopamine D1 receptors in the dentate gyrus in antidepressant actions of a selective serotonin reuptake inhibitor, fluoxetine
Source: Mol Psychiatry. 2018 Dec 10;25(6):1229–44. doi: 10.1038/s41380-018-0316-x (PMC7244404; doi:10.1038/s41380-018-0316-x)
Supplement: Supplementary file 8 — Supplementary Figure 8 [file 41380_2018_316_MOESM8_ESM.pptx]

## Slide 1
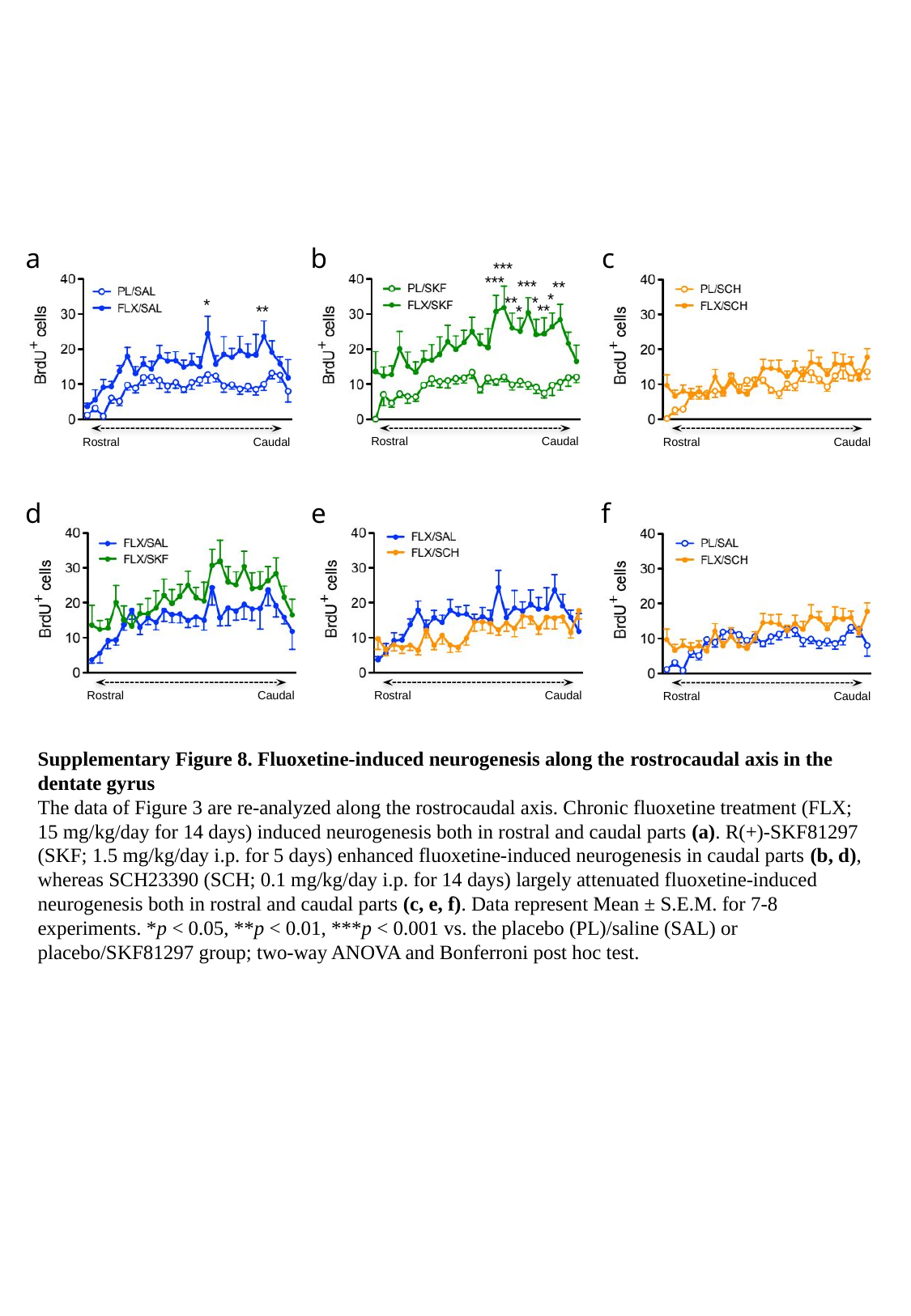

a
Rostral
Caudal
b
Rostral
Caudal
c
Rostral
Caudal
***
***
***
**
*
**
*
*
**
*
**
d
Rostral
Caudal
e
Rostral
Caudal
f
Rostral
Caudal
Supplementary Figure 8. Fluoxetine-induced neurogenesis along the rostrocaudal axis in the dentate gyrus
The data of Figure 3 are re-analyzed along the rostrocaudal axis. Chronic fluoxetine treatment (FLX; 15 mg/kg/day for 14 days) induced neurogenesis both in rostral and caudal parts (a). R(+)-SKF81297 (SKF; 1.5 mg/kg/day i.p. for 5 days) enhanced fluoxetine-induced neurogenesis in caudal parts (b, d), whereas SCH23390 (SCH; 0.1 mg/kg/day i.p. for 14 days) largely attenuated fluoxetine-induced neurogenesis both in rostral and caudal parts (c, e, f). Data represent Mean ± S.E.M. for 7-8 experiments. *p < 0.05, **p < 0.01, ***p < 0.001 vs. the placebo (PL)/saline (SAL) or placebo/SKF81297 group; two-way ANOVA and Bonferroni post hoc test.
