# Supplementary material for: Obligatory roles of dopamine D1 receptors in the dentate gyrus in antidepressant actions of a selective serotonin reuptake inhibitor, fluoxetine
Source: Mol Psychiatry. 2018 Dec 10;25(6):1229–44. doi: 10.1038/s41380-018-0316-x (PMC7244404; doi:10.1038/s41380-018-0316-x)
Supplement: Supplementary file 9 — Supplementary Figure 9 [file 41380_2018_316_MOESM9_ESM.pptx]

## Slide 1
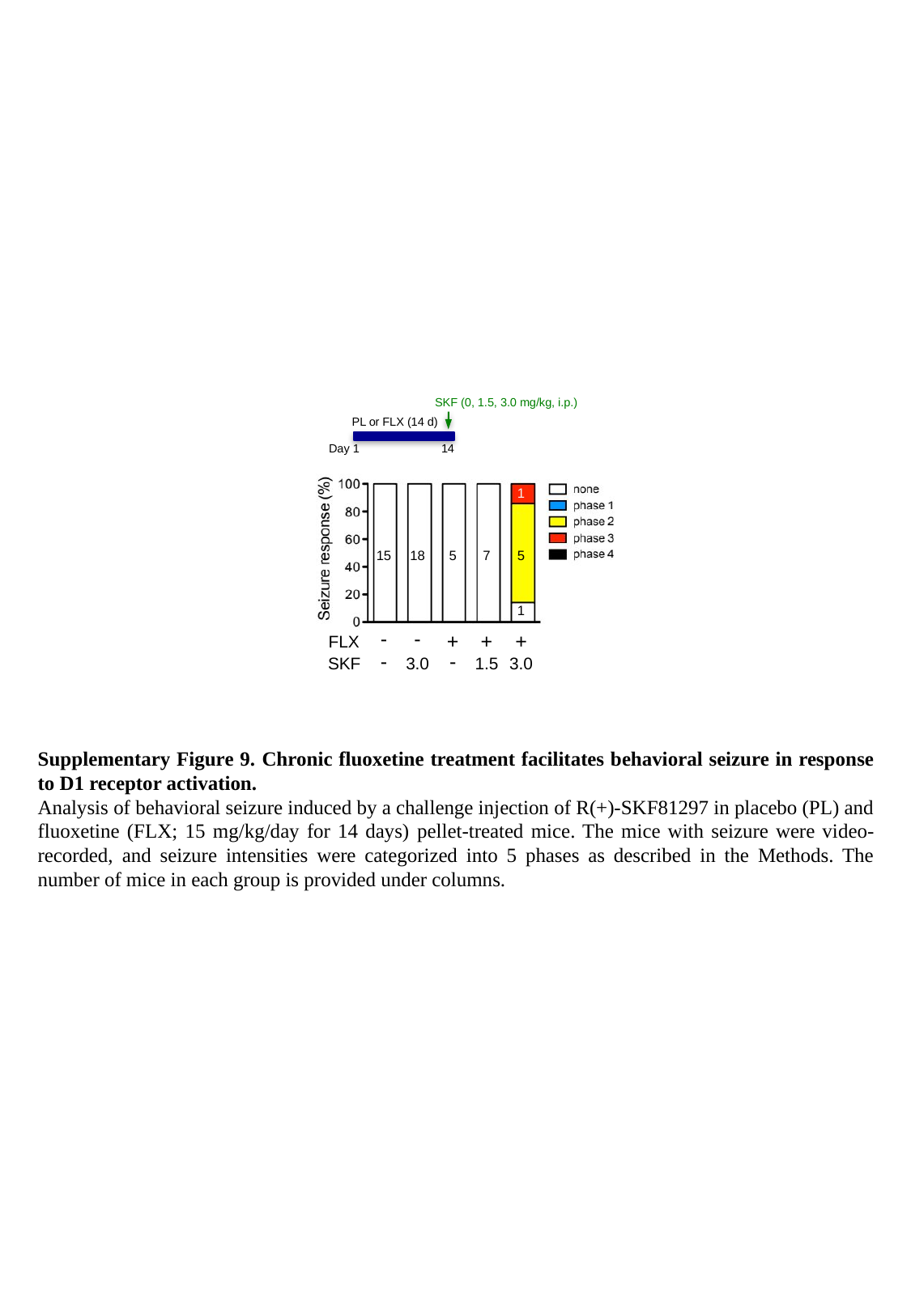

SKF (0, 1.5, 3.0 mg/kg, i.p.)
PL or FLX (14 d)
Day 1
14
1
15
18
5
7
5
1
-
-
+
+
+
FLX
-
-
SKF
3.0
1.5
3.0
Supplementary Figure 9. Chronic fluoxetine treatment facilitates behavioral seizure in response to D1 receptor activation.
Analysis of behavioral seizure induced by a challenge injection of R(+)-SKF81297 in placebo (PL) and fluoxetine (FLX; 15 mg/kg/day for 14 days) pellet-treated mice. The mice with seizure were video-recorded, and seizure intensities were categorized into 5 phases as described in the Methods. The number of mice in each group is provided under columns.
