# Supplementary material for: Obligatory roles of dopamine D1 receptors in the dentate gyrus in antidepressant actions of a selective serotonin reuptake inhibitor, fluoxetine
Source: Mol Psychiatry. 2018 Dec 10;25(6):1229–44. doi: 10.1038/s41380-018-0316-x (PMC7244404; doi:10.1038/s41380-018-0316-x)
Supplement: Supplementary file 10 — Supplementary Figure 10 [file 41380_2018_316_MOESM10_ESM.pptx]

## Slide 1
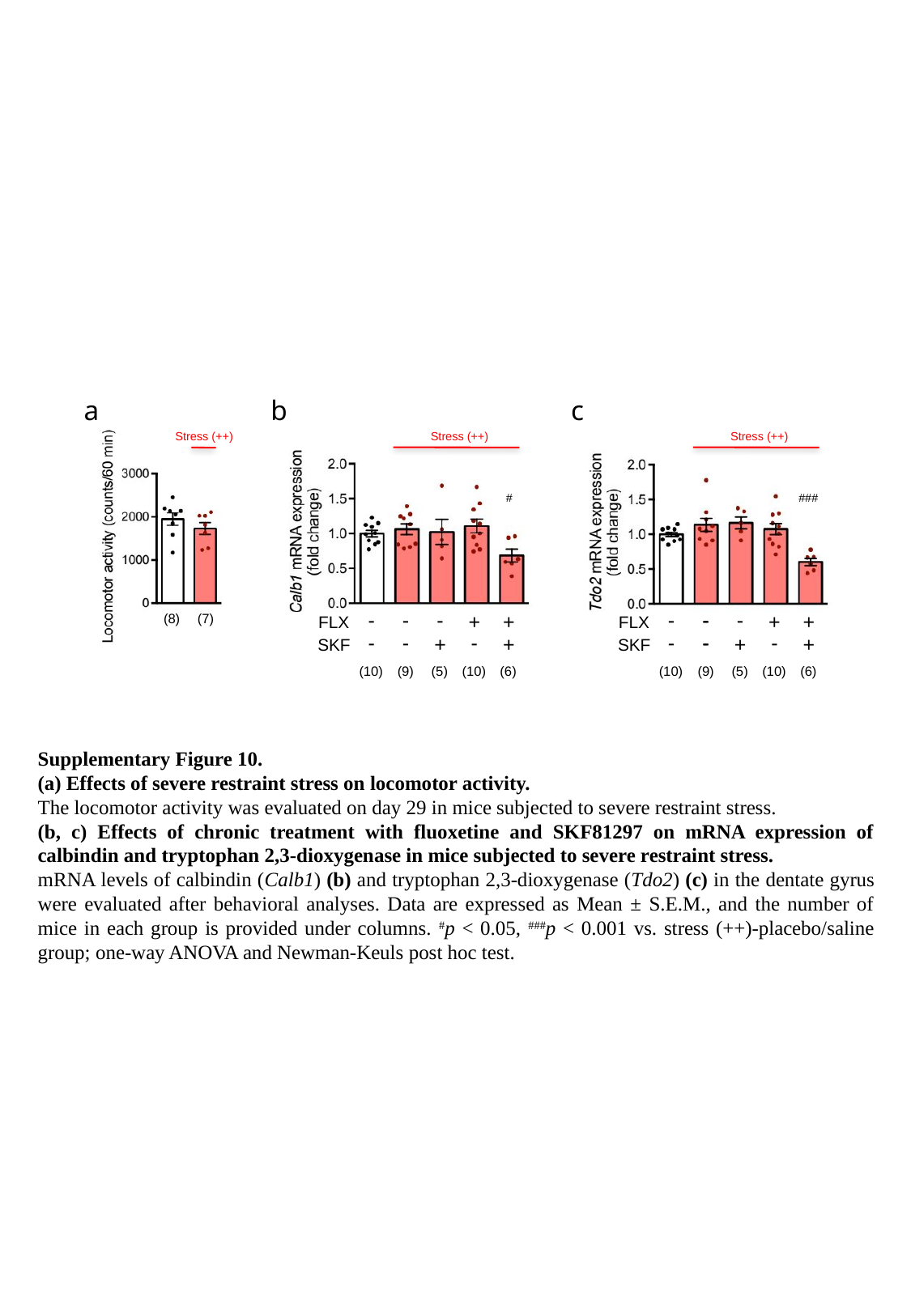

a
b
c
Stress (++)
Stress (++)
Stress (++)
#
###
-
-
-
-
-
-
-
+
+
+
+
(8)
(7)
FLX
FLX
-
-
-
-
-
-
-
+
+
+
+
SKF
SKF
(10)
(9)
(5)
(10)
(6)
(10)
(9)
(5)
(10)
(6)
Supplementary Figure 10.
(a) Effects of severe restraint stress on locomotor activity.
The locomotor activity was evaluated on day 29 in mice subjected to severe restraint stress.
(b, c) Effects of chronic treatment with fluoxetine and SKF81297 on mRNA expression of calbindin and tryptophan 2,3-dioxygenase in mice subjected to severe restraint stress.
mRNA levels of calbindin (Calb1) (b) and tryptophan 2,3-dioxygenase (Tdo2) (c) in the dentate gyrus were evaluated after behavioral analyses. Data are expressed as Mean ± S.E.M., and the number of mice in each group is provided under columns. #p < 0.05, ###p < 0.001 vs. stress (++)-placebo/saline group; one-way ANOVA and Newman-Keuls post hoc test.
