# Supplementary material for: Obligatory roles of dopamine D1 receptors in the dentate gyrus in antidepressant actions of a selective serotonin reuptake inhibitor, fluoxetine
Source: Mol Psychiatry. 2018 Dec 10;25(6):1229–44. doi: 10.1038/s41380-018-0316-x (PMC7244404; doi:10.1038/s41380-018-0316-x)
Supplement: Supplementary file 11 — Supplementary Figure 11 [file 41380_2018_316_MOESM11_ESM.pptx]

## Slide 1
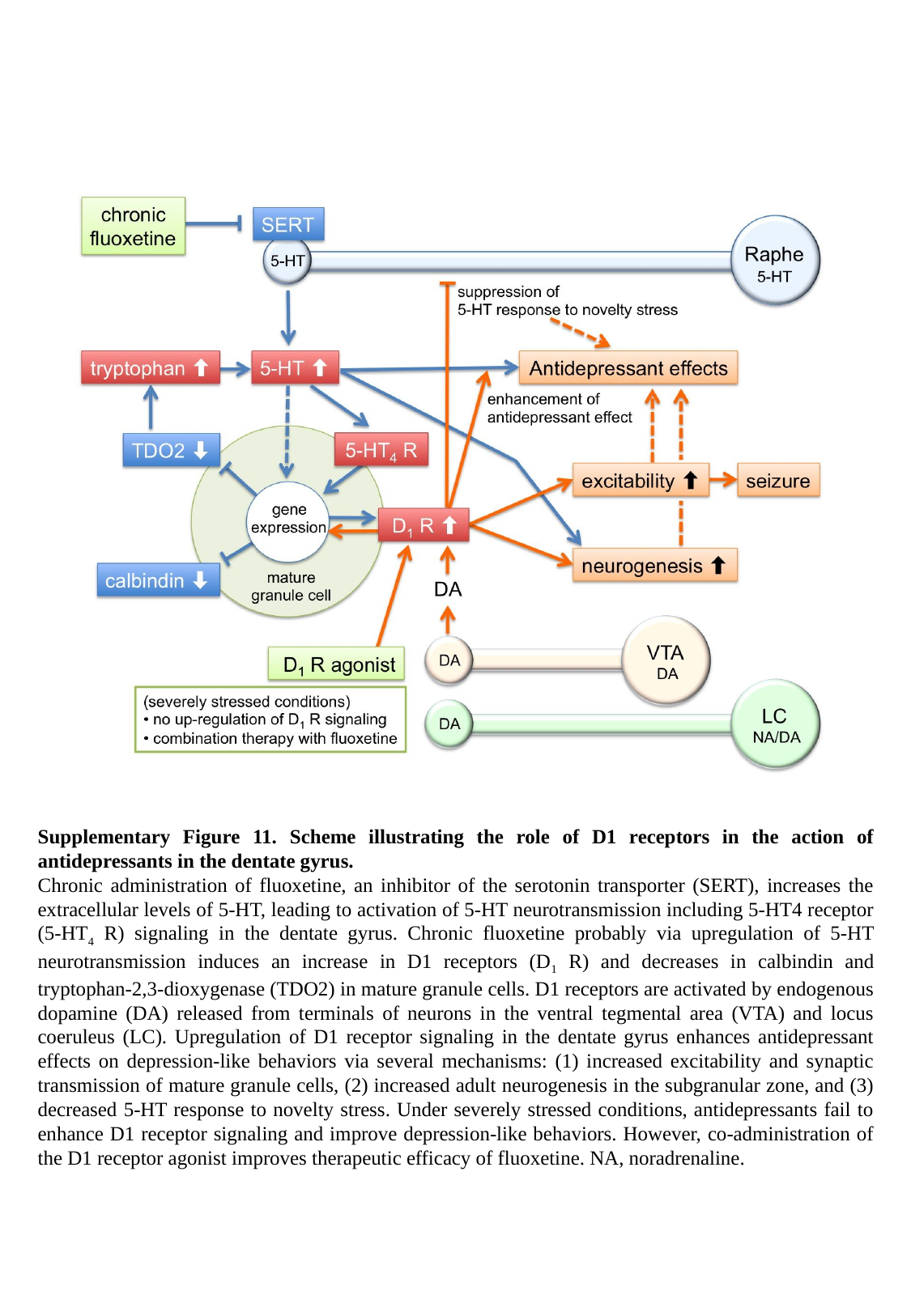

Supplementary Figure 11. Scheme illustrating the role of D1 receptors in the action of antidepressants in the dentate gyrus.
Chronic administration of fluoxetine, an inhibitor of the serotonin transporter (SERT), increases the extracellular levels of 5-HT, leading to activation of 5-HT neurotransmission including 5-HT4 receptor (5-HT4 R) signaling in the dentate gyrus. Chronic fluoxetine probably via upregulation of 5-HT neurotransmission induces an increase in D1 receptors (D1 R) and decreases in calbindin and tryptophan-2,3-dioxygenase (TDO2) in mature granule cells. D1 receptors are activated by endogenous dopamine (DA) released from terminals of neurons in the ventral tegmental area (VTA) and locus coeruleus (LC). Upregulation of D1 receptor signaling in the dentate gyrus enhances antidepressant effects on depression-like behaviors via several mechanisms: (1) increased excitability and synaptic transmission of mature granule cells, (2) increased adult neurogenesis in the subgranular zone, and (3) decreased 5-HT response to novelty stress. Under severely stressed conditions, antidepressants fail to enhance D1 receptor signaling and improve depression-like behaviors. However, co-administration of the D1 receptor agonist improves therapeutic efficacy of fluoxetine. NA, noradrenaline.
